# Explainable Graph Spectral Clustering of text documents

**DOI:** 10.1371/journal.pone.0313238

**Published:** 2025-02-04

**Authors:** Bartłomiej Starosta, Mieczysław A. Kłopotek, Sławomir T. Wierzchoń, Dariusz Czerski, Marcin Sydow, Piotr Borkowski

**Affiliations:** 1 Institute of Computer Science, Polish Academy of Sciences, Warsaw, Poland; 2 Polish-Japanese Academy of Information Technology, Warsaw, Poland; Universidade de Sao Paulo, BRAZIL

## Abstract

Spectral clustering methods are known for their ability to represent clusters of diverse shapes, densities etc. However, the results of such algorithms, when applied e.g. to text documents, are hard to explain to the user, especially due to embedding in the spectral space which has no obvious relation to document contents. Therefore, there is an urgent need to elaborate methods for explaining the outcome of the clustering. We have constructed in this paper a theoretical bridge linking the clusters resulting from Graph Spectral Clustering and the actual document content, given that similarities between documents are computed as cosine measures in tf or tfidf representation. This link enables to provide with explanation of cluster membership in clusters produced by GSA. We present a proposal of explanation of the results of combinatorial and normalized Laplacian based graph spectral clustering. For this purpose, we show (approximate) equivalence of combinatorial Laplacian embedding and of *K*-embedding (proposed in this paper) and term vector space embedding. We performed an experimental study showing that *K*-embedding approximates well Laplacian embedding under favourable block matrix conditions and show that approximation is good enough under other conditions. We show also perfect equivalence of normalized Laplacian embedding and the M-embedding (proposed in this paper) and (weighted) term vector space embedding. Hence a bridge is constructed between the textual contents and the clustering results using both combinatorial and normalized Laplacian based Graph Spectral Clustering methods. We provide a theoretical background for our approach. An initial version of this paper is available at arXiv, (Starosta B 2023). The Reader may refer to that text to get acquainted with formal aspects of our method and find a detailed overview of motivation.

## 1 Introduction

We propose a theoretical bridge linking the clusters resulting from Graph Spectral Clustering and the actual document content, given that similarities between documents are computed as cosine measures in *tf* or *tfidf* representation. This link enables us to provide the explanation of cluster membership in clusters produced by GSA. We provide textual justification for a document’s cluster membership derived from cosine similarity, and at the same time provide textual justification for its non-membership in other clusters via distance computation in the document vector embedding space. This result is novel as various authors recommend not to use GSA if you “need an explainable algorithm”. See e.g. https://crunchingthedata.com/when-to-use-spectral-clustering/.

The reason for focusing our research on the so-called graph spectral cluster analysis (GSA) in application to sparse datasets stems from the fact that GSA is applicable for high-dimensional datasets. This is a relatively rare feature among existing clustering algorithms. GSA clustering methods are known for their ability to represent clusters of diverse shapes, different densities, various cardinalities etc. They constitute an approximation to graph cuts of various types (plain cuts, normalized cuts, and ratio cuts). Further, they apply to unweighted and weighted similarity graphs. Despite their advantages, various shortcomings were encountered in their application. The need for computing eigenvectors makes it relatively slow. Like *k*-means, they are sensitive to the initialization conditions that are used. What seems to be most painful, is that it is hard to explain results. One of the most frequently applied clustering algorithms, *k*-means algorithm behaves perfectly when data are low dimensional and can be divided into spherical clusters of similar cardinality. The results are easy to explain then. However, the reality of textual documents is that the clusters are usually not spherical, of different cardinality, and the dimensionality is high, up to dozens of thousands of dimensions. Therefore approaches like GSA are considered.

Explainable AI (XAI) is an important developing area but remains relatively understudied for clustering. One expects that the information within a cluster represents a coherent piece of knowledge. However, in reality, we need a human inspection stage to understand cluster content, in order to answer the question: “What is this cluster about?”. This need for human intervention limits the use of clusters in automatic decision-making processes. An automatic explanation of cluster content could remove this human step, such that the users and/or application would be enabled to focus on consuming the clustering results. The extraction of information that explains the semantic content of the clusters is still mostly a manual activity, as it requires the inspection of sample documents [1, 2].

Notably, the core of GSA is usually a *k*-means algorithm, possessing a multitude of cluster explanation methods, see [3–7]. In spite of this, the result of GSA clustering is hard to explain to the user due to the embedding of clusters in the spectral space, that has no direct relation to the document texts. This forces the users to examine manually the clusters to gain insights which may turn out to be time-consuming. Typically, it is recommended *not to use* spectral clustering if you “need an explainable algorithm”. See e.g. https://crunchingthedata.com/when-to-use-spectral-clustering/.

The problem is that GSA describes the clusters in terms of values of eigenvectors. But what the people need is the description in human terms. This paper seeks to overcome this weakness. We devise an automated method to describe/explain GSA clusters in terms of natural language words. This shall enable the users and/or application to focus just on consuming the clustering results that fit their needs.

This is of practical importance as GSA is used frequently in the context of natural language processing, see e.g. [8–11]. We propose an explanation method of the results of two types of GSA clustering algorithms: one based on combinatorial Laplacian (we shall call this GSA clustering method *L-based clustering*) the other based on normalized Laplacian (we shall call this GSA clustering method *N-based clustering*, *N* standing for “normalized”). *L*-based clustering is an approximation to the graph clustering method called RCut and *N*-based clustering is an approximation to the graph clustering method called NCut [12]. Our approach to explainability is as follows: We propose an alternative “theoretical” clustering method, that we call *k-based clustering* and demonstrate its approximate equivalence to RCut and hence *L*-based clustering. Then we recall the traditional term-vector-space clustering method (*TVS-based*) and demonstrate that it is equivalent to *k*-based clustering. But the *TVS*-based clustering is easily explained in terms of words stemming from documents. So we have provided in this way explainability to combinatorial Laplacian based GSA clustering. On the other hand, to explain the results of normalized Laplacian GSA based clustering, we propose an alternative “theoretical” clustering method, that we call M-*based clustering* and demonstrate its full equivalence to NCut and hence approximation to *N*-based clustering. Then we introduce a special, new weighted term-vector-space clustering method (*weighted TVS-based*) and demonstrate that it is equivalen t to M-based clustering. We provide also a prescription for how the weighted *TVS*-based clustering can be explained in terms of words stemming from documents.

Thanks to these results, we can explain cluster membership of textual documents in Laplacian embedding by pointing at significant words/terms, as commonly practised [13], subject to various improvements [14, 15] (compare also with Shapley-value based approaches [16]). Note that other hard-to-explain clusterings like those generated by deep methods [17], short-text based user clustering [18], use this form of explanation. Before presenting our method, we review in Section 2 previous research on clustering explanations. Section 3 provides a brief overview of GSA, in particular of combinatorial and normalized Laplacian based clustering. Section 4 explains the relationship between GSA and graph cut clustering methods. In particular, Section 4.1 recalls the relationship between combinatorial Laplacian based clustering and RCut, while Section 4.2 reminds the relationship between normalized Laplacian based clustering and NCut. Section 5 introduces our proposal for the explanation of combinatorial Laplacian based (*L*-based), and Section 6—normalized Laplacian based (*N*-based) spectral clustering. In particular, Section 5.1 explains our alternative (*K*-based) clustering method. Section 5.2 shows that *K*-based clustering method approximates the target combinatorial GSA. Section 5.4 shows that *K*-based clustering method is equivalent to clustering in Term Vector Space. As normalized GSA is concerned, we introduce in Section 6.1 our new (M-based) clustering method. Section 6.2 shows that M-based clustering method approximates the target normalized GSA. Section 6.4 shows that M-based clustering method is equivalent to clustering in weighted Term Vector Space.

Section 7 presents some experimental results on clustering using the combinatorial Laplacian based clustering and our *K*-embedding based clustering. We conclude the paper with some final remarks in Section 8.

The Reader may refer to the initial version of this paper, available at arXiv, [19], in order to get acquainted with formal aspects of our method and find a detailed overview of motivation.

## 2 Previous research

As the realm of clustering algorithms is vast, see e.g. [20–23], we narrow our interest to the large family of spectral clustering algorithms [12, 24–26], which have numerous desirable properties (like detection of clusters with various shapes, applicability to high dimensional datasets, and capability to handle categorical variables). But, as mentioned, they are not free from various shortcomings, common to other sets of algorithms, including multiple possibilities of representation of the same dataset, producing results in a space different from the space of the original problem, curse of dimensionality etc., which are particularly grieving under large and sparse dataset scenario.

Cluster Analysis, like the entire domain of Artificial Intelligence, experienced rapid development over the recent years, providing algorithms of growing complexity and efficiency that are regrettably characterized by their “black-box nature” that is their results are hard to understand by human users and therefore there exists a growing resistance for their application in practical settings. This phenomenon led to the development of a branch of AI called “Explainable Artificial Intelligence” (XAI) [27, 28], with subbranches including Explainable Clustering [29]. A very general overview of methods of explainable Artificial Intelligence is given by [30]. It has to be mentioned, that short documents have some challenging peculiarities, as [31] shows.

The “black box” problem relates in particular to cluster analysis [29]. The situation is more difficult here, compared e.g. to the classification tasks, because the very essence of the concept of “cluster” is not well-defined. Even though, the scientific research area of cluster analysis has nearly a century-long history, during which hundreds of clustering algorithms have been developed, and countless applications are reported.

Recent years have brought visible progress in this area. Multiple explainable versions of *k*-means algorithm have been elaborated, [3–5] with multiple improvements [32, 33] and applications, e.g. [6, 7]. They generate explanations in terms of the underlying features used in the clustering, which is not usable in GSA as the features here are not human-interpretable.

[34] propose a different, exemplar-based approach to clustering explanation which may be suitable for various embedding types, like auto-encoders or word embeddings. As selecting a small set of exemplars to explain even a single cluster appears to be computationally intractable, they developed an approximation algorithm. Its basic version explains all the instances in every cluster, while an extension detects a bounded number of exemplars providing explanations covering a large fraction of all the instances. [35] presents a similar idea, but rather based on prototypes. [2] concentrate on explanations via relevant keywords.

While the mentioned methods are not well suited for explaining text clustering, authors of [36] suggest a quite universal method for text cluster explanation. The method is based on creating an equivalent neural network model for a given clustering of text documents. The network shall be trained via backpropagation. Then, via backpropagation too, the words determining a given cluster membership can be read out. In a similar spirit, but much more elaborated, taking into account also semantic features, the authors of [17] propose a deep neural network suitable for both clustering and its explanation. Notably, in both approaches, the explanation provided is in form of a list of word/phrases characterizing each cluster. Authors of [37] concentrate on the deep models enriched with multi-view aspects. Still another deep learning approach in [38] exploits the concept of self-organizing-maps.

Authors of [18] use a (hidden variable) probabilistic model with the detection of hidden topics generating word pairs to perform clustering into topics and then to describe the topics by the distribution of word pairs implied by the topic.

Authors of [1] stress the need to define appropriate similarity measures as clusters need to contain similar documents.

## 3 A brief overview of Graph Spectral Clustering

Throughout this paper, we will use the symbols listed in Table 1.

**Table 1:** Symbols and abbreviations used throughout the paper.

Symbol	meaning
GSA	Graph Spectral Clustering
D	the set of documents
*n*	the number of documents
*C*_*j*_	the set of elements of a document cluster *j*
*n*_*j*_	the number of elements in the document cluster *j*
*S*	similarity matrix, *s*_*iℓ*_—similarity between document *i* and *ℓ*; the diagonal elements *s*_*ii*_ are all equal to zero.
*D*	diagonal matrix containing row sums of the similarity matrix *S*; *d*_*ii*_—the element *i* of the diagonal of *D*; *ω*_*i*_ = *d*_*ii*_—the weight of document *i*, when performing weighted clustering.
*L*	combinatorial Laplacian of the similarity matrix *S*; see formula (1)
L	normalized Laplacian of the similarity matrix *S*; see formula (2)
*Q*^[*RCut*]^(Γ)	the clustering quality criterion (for clustering Γ) for RCut clustering algorithm; see formula (3)
*Q*^[*NCut*]^(Γ)	the clustering quality criterion (for clustering Γ) for NCut clustering algorithm; see formula (11)
*Q*^[*GSAL*]^(Γ)	the clustering quality criterion (for clustering Γ) for GSA clustering algorithm based on combinatorial Laplacian *L*; see formula (9)
$Q^{\left[GSAL\right]}\left(\Gamma \right)$	the clustering quality criterion (for clustering Γ) for GSA clustering algorithm based on normalized Laplacian *L*; see formula (17)
*Q*^[*Kbased*]^(Γ)	the clustering quality criterion (for clustering Γ) for *K*-based clustering algorithm; see formula (22)
$Q^{\left[Mbased\right]}\left(\Gamma ;\omega \right)$	the clustering quality criterion (for clustering Γ) for M-based clustering algorithm; see formula (39)
*Q*^[*TVS*]^(Γ)	the clustering quality criterion (for clustering Γ) for clustering algorithm in Term Vector Space, approximating RCut; see formula (25)
*Q*^[*ωTVS*]^(Γ; ***ω***)	the clustering quality criterion (for clustering Γ) for clustering algorithm in weighted Term Vector Space, equivalent to NCut; see formula (58)
**x**_*i*_	vector representing document *i* in the space spanned by eigenvectors of combinatorial Laplacian *L*
***ξ***_*i*_	vector representing document *i* in the space spanned by eigenvectors of normalized Laplacian L
**z**_*i*_	vector representing document *i* in the space generated by the *K*-embedding
***ζ***_*i*_	vector representing document *i* in the space generated by the M-embedding
**w**_*i*_	vector representing document *i* in the Term Vector Space.
**w**′_*i*_	vector representing document *i* in the weighted Term Vector Space.

Graph spectral clustering methods can be viewed as a relaxation of cut based graph clustering methods. Let *S* be a (symmetric) similarity matrix between pairs of items (e.g. documents). It induces a graph whose nodes correspond to the items (documents). In the domain of text mining, the similarity matrix is usually based on either a graph representation of relationships (links) between items (text documents) or such a graph is induced by (cosine) similarity measures between items (document texts). However, mixed object representations (text and links) have also been studied [39]. By convention, all diagonal elements of matrix *S* are equal to zero.

A(n unnormalised or) combinatorial Laplacian *L* corresponding to this matrix (approximating the RCut) is defined as

$$\begin{array}{c} L=D-S, \end{array}$$ (1)

where *D* is the diagonal matrix with $d_{ii}=\sum_{\ell =1}^{n}s_{i\ell }$ for each *i* ∈ [*n*]. A normalized Laplacian L of the graph represented by *S* (approximating NCut) is defined as

$$\begin{array}{c} L=D^{-1/2}LD^{-1/2}=I-D^{-1/2}SD^{-1/2}. \end{array}$$ (2)

The power operations performed on the diagonal matrix *D*^*x*^ are not real matrix power operations. They are rather performed on each element of the diagonal of *D* separately, not affecting the zeros outside of the diagonal (*D*′ = *D*^*x*^ means computing $d_{ii}^{\prime }=d_{ii}^{x}$ for each diagonal element of *D*, and setting $d_{i\ell }^{\prime }=0$ for each off-diagonal element.) In particular, *D*^−1^ is a pseudo-inverse of *D*.

Whichever Laplacian is used, the clustering is performed as follows. We assume that we want to cluster the data into *k* clusters. One computes the eigen-decomposition of the Laplacian, getting *n* eigenvalues λ_1_ ≤ ⋯ ≤ λ_*n*_ (always λ_1_ = 0) and corresponding eigenvectors **v**_1_, …, **v**_*n*_. Then one embeds the documents in the *k*-dimensional space spanned by the *k* eigenvectors corresponding to *k* lowest eigenvalues. That is, one assigns each document *i* the coordinates [*v*_*i*,1_, …, *v*_*i*,*k*_]. This shall be called *L*-embedding if the combinatorial Laplacian *L* is used, and *N*-embedding, if the normalized Laplacian L is used. Then one clusters the documents in this embedding using e.g. *k*-means algorithm.

Let us briefly recall the typical spectral clustering algorithm in order to make it understandable, how distant the clustering may be from the applier’s comprehension [12]. The first step consists in creating a similarity matrix of objects (in case of documents based on tf, tfidf, in unigram or *n*-gram versions, or some transformer based embeddings are the options—consult e.g. [40] for details), then mixing them in case of multiple views available. The second step is to calculate a Laplacian matrix. There are at least three variants to use: combinatorial, normalized, and random-walk Laplacian, [12]. Then computing eigenvectors and eigenvalues, eigenvector smoothing (to remove noise and/or achieve robustness against outliers) choice of eigenvectors, and finally clustering in the space of selected eigenvectors (via e.g. *k*-means).

Detailed descriptions can be found e.g. in [12, 21]. When the clustering is finalized, then for each item *i* we have its coordinates [*v*_*i*,1_, …, *v*_*i*,*k*_] and its membership in some cluster *C*_*j*_, but we cannot tell why *i* belongs to *C*_*j*_ because none of the coordinates [*v*_*i*,1_, …, *v*_*i*,*k*_] has anything to do with the contents of the document *i*, in particular with its term frequency (tf, tfidf) or any other content representation. Therefore our goal is to find a justified way to tell which terms are the reason for cluster membership of a document.

## 4 The Graph Spectral Clustering versus graph cuts

The relationship between Graph Spectral Clustering methods and Graph Cut methods is as follows: The RCut criterion corresponds to finding the partition matrix $P_{RCut}\in R^{n\times k}$ that minimizes the formula *H*′*LH* (where H’ stands for the transpose of H) over the set of all partition matrices $H\in R^{n\times k}$. Such a formulated problem is NP-hard. That is why we relax it by assuming that *H* is a column orthogonal matrix. In this case the solution is obvious: the columns of *P*_*RCut*_ are eigenvectors of *L* corresponding to *k* smallest eigenvalues of *L*. Similarly, the columns of matrix *P*_*NCut*_, representing NCut criterion, are eigenvectors of L corresponding to *k* smallest eigenvalues of L. We provide more details in the subsequent subsections. For an in-depth explanation and further details see e.g. [12, 21].

### 4.1 The goal of Graph Spectral Clustering versus RCut

The RCut clustering aims at splitting the dataset into *k* clusters Γ = {*C*_1_, …, *C*_*k*_} minimizing the following criterion:

$$\begin{array}{c} Q^{\left[RCut\right]}\left(\Gamma \right)=\sum_{j=1}^{k}\frac{1}{n_{j}}\sum_{i\in C_{j}}\sum_{\ell \notin C_{j}}s_{i\ell } \end{array}$$ (3)

where *n*_*j*_ = |*C*_*j*_|, that is we minimize the sum over all clusters of sum of similarity between elements of a given cluster and other clusters divided by the cluster cardinality.

Let us reformulate this task, following the ideas of Hall [41]. Imagine, we want to embed the set of documents D={1,…,n} in an Euclidean space $R^{k}$ in such a way that the clusters in this space reflect the clustering via RCut criterion. This embedding shall be denoted by a matrix *Y* such that *y*_*ij*_ indicates the membership of document *i* in cluster *j*. In the Euclidean space mentioned above, the row vector of this matrix (*y*_*i*1_, …, *y*_*ik*_) means coordinates of document *i* in the space $R^{k}$ and the column vector **y**_*j*_ is the indicator vector of class membership of the cluster *j*. Let

$$\begin{array}{cc} y_{ij}=\frac{1}{\sqrt{n_{j}}}\;, & \text{if}\;\text{the}\;\text{document}\;i\;\text{belongs}\;\text{to}\;\text{cluster}\;j\\ y_{ij}=0 & \text{otherwise} \end{array}$$ (4)

Note that under this definition, all indicator vectors are of unit length, ‖**y**_*j*_‖ = 1, and are mutually orthogonal ($y_{j}^{T}y_{j^{\prime }}=0$, for *j*′ ≠ *j*). Hall [41] proposed the following criterion to be minimized when embedding a graph in Euclidean space:

$$\begin{array}{c} E_{H}\left(Y;S\right)=\frac{1}{2}\sum_{i=1}^{n}\sum_{\ell =1}^{n}(\sum_{j=1}^{k}{\left(y_{ij}-y_{\ell j}\right)}^{2})s_{i\ell } \end{array}$$ (5)

It is also worth noting that we can reexpress the above Hall criterion as

$$\begin{array}{c} E_{H}\left(Y;S\right)=\sum_{j=1}^{k}y_{j}^{T}Ly_{j} \end{array}$$ (6)

where *L* is the combinatorial Laplacian of the similarity matrix *S*. It can be immediately seen that given the above definitions of *y*_*ij*_

$$\begin{array}{c} E_{H}\left(Y;S\right)=Q^{\left[RCut\right]}\left(\Gamma \right) \end{array}$$ (7)

This means that minimizing the Hall criterion (while maintaining the constraints imposed on the indicator vectors) is equivalent to minimizing the RCut criterion when clustering the data.

Note also that under the aforementioned Hall embedding, documents of the same cluster are located at the same point in the Euclidean space so that any algorithm, and in particular the *k*-means algorithm easily finds the clusters.

As mentioned, the minimization of the formula (6) under the constraint (4) is NP-hard.

This is the point where GSA provides a solution. The constraint (4) is replaced with the mere requirement that the matrix *Y* is real-valued and that vectors **y**_*j*_ are of unit length and orthogonal to each other. Under this relaxation, by the Rayleigh-Ritz theorem, the minimization of (6) can be reduced to solving the eigen-decomposition problem of *L*. If we additionally require that vectors **y**_*j*_ are different from each other and vector entries are not the same at every coordinate, then

$$\begin{array}{c} \underset{Y}{\text{min}}\;\left(E_{H}\left(Y;S\right)\right)=\lambda_{2}+\cdots +\lambda_{k+1} \end{array}$$ (8)

where λ_1_ = 0, λ_2_, …, λ_*k*+1_ are the lowest eigenvalues of *L* (sorted non-decreasingly), with eigenvectors corresponding to λ_2_, …, λ_*k*+1_ being the sought indicator vectors $y_{j}^{\prime }$. The clustering performed in this embedding approximates therefore the clustering Γ that minimizes RCut.

So *L*-based GSA minimizes the criterion

$$\begin{array}{c} Q^{\left[GSAL\right]}\left(\Gamma \right)=\sum_{j=1}^{k}\sum_{i\in C_{j}}\left|\right|x_{i}-\mu \left(C_{j}\right){\left|\right|}^{2} \end{array}$$ (9)

where ***μ***(*C*_*j*_) is the center of cluster *C*_*j*_, equal to:

$$\begin{array}{c} \mu \left(C_{j}\right)=\frac{1}{|C_{j}|}\sum_{i\in C_{j}}x_{i} \end{array}$$ (10)

whereby **x**_*i*_ = (*y*′_*i*2_, …, *y*′_*i*,*k*+1_)^*T*^, and **y**′_1_ is the eigenvector of *L* corresponding to its eigenvalue λ_2_, **y**′_2_ to λ_3_ and so on.

We shall call this embedding **x**_*i*_ of documents *i* from D the *L-based embedding*.

The formula (9) is exactly the target function of the *k*-means algorithm in the respective (Euclidean) embedding space. It is hence quite natural that its minimum is sought using the traditional *k*-means algorithm.

Note that if the eigenvectors of *L* were really the **y**_*j*_ indicator vectors, then *k*-means would achieve the absolute minimum equal to zero and return the intrinsic RCut clustering (and RCut optimum would be reached).

The disadvantage of *L*-embedding **x**_*i*_ is that there is no direct link between its components and the cosine similarity between textual documents from the collection D. Hence, an explanation of cluster membership is not straightforward. In the subsequent section 5 we seek a way out of this situation.

### 4.2 The goal of Graph Spectral Clustering versus NCut

The NCut clustering aims at splitting the dataset into *k* clusters Γ = {*C*_1_, …, *C*_*k*_} minimizing the following criterion:

$$\begin{array}{c} Q^{\left[NCut\right]}\left(\Gamma \right)=\sum_{j=1}^{k}\frac{1}{V_{j}}\sum_{i\in C_{j}}\sum_{\ell \notin C_{j}}s_{i\ell } \end{array}$$ (11)

where $V_{j}=\sum_{i\in C_{j}}d_{ii}$, that is we minimize the sum over all clusters of sum of similarity between elements of a given cluster and other clusters divided by the cluster volume $V_{j}$.

Let us reformulate this task, following the ideas of Belkin & Niyogi [42]. Imagine, we want to embed the set of documents D={1,…,n} in an Euclidean space $R^{k}$ in such a way that the clusters in this space reflect the clustering via NCut criterion. This embedding shall be denoted by a matrix Υ such that *υ*_*ij*_ is the indicator of the membership of document *i* in cluster *j*. In the Euclidean space mentioned above, the row vector of this matrix (*υ*_*i*1_, …, *υ*_*ik*_) means coordinates of document *i* in the aforementioned space $R^{k}$ and the column vector ***υ***_*j*_ is the indicator vector of class membership of the cluster *j*. Let

$$\begin{array}{cc} \upsilon_{ij}=\sqrt{\frac{d_{ii}}{V_{j}}}\;, & \text{if}\;\text{the}\;\text{document}\;i\;\text{belongs}\;\text{to}\;\text{cluster}\;j\\ \upsilon_{ij}=0 & \text{otherwise} \end{array}$$ (12)

Note that under this definition, all indicator vectors are of unit length, ‖***υ***_*j*_‖ = 1, and are pairwise orthogonal ($\upsilon_{j}^{T}\upsilon_{j^{\prime }}=0$, for *j*′ ≠ *j*). Let us define, following Belkin & Niyogi [42], the embedding criterion, to be minimized:

$$\begin{array}{c} E_{B}\left(\Upsilon ;S\right)=\frac{1}{2}\sum_{i=1}^{n}\sum_{\ell =1}^{n}(\sum_{j=1}^{k}{\left(\frac{\upsilon_{ij}}{\sqrt{d_{ii}}}-\frac{\upsilon_{\ell j}}{\sqrt{d_{\ell \ell }}}\right)}^{2})s_{i\ell } \end{array}$$ (13)

It is also worth noting that we can reexpress the above Belkin & Niyogi criterion as

$$\begin{array}{c} E_{B}\left(Y;S\right)=\sum_{j=1}^{k}\upsilon_{j}^{T}L\upsilon_{j} \end{array}$$ (14)

where L is the normalized Laplacian of the similarity matrix *S*. It can be immediately seen that given the above definitions of *υ*_*ij*_ that

$$\begin{array}{c} E_{B}\left(\Upsilon ;S\right)=Q^{\left[NCut\right]}\left(\Gamma \right) \end{array}$$ (15)

This means that minimizing Belkin & Niyogi criterion (while keeping the constraints imposed on indicator vectors) means minimizing NCut criterion when clustering data.

Note also that under the aforementioned Belkin & Niyogi embedding, documents of the same cluster are located at the same point in the Euclidean space so that any algorithm and in particular *k*-means algorithm easily finds the clusters.

The problem is, however, that minimization of formula (14) under the constraint (12) is NP-hard.

This is the point where GSA provides with a solution, in a way analogous to that on page 7. The constraint (12) is relaxed to requiring only that the matrix Υ is real-valued and that vectors ***υ***_*j*_ are of unit length and orthogonal to each other. Given this assumption, by the Rayleigh-Ritz theorem, the minimization of (14) means solving the eigen-decomposition problem of L. Under the additional requirement, that vectors ***υ***_*j*_ are different from each other and are not same at each coordinate, we have

$$\begin{array}{c} \underset{\Upsilon }{\text{min}}\;\left(E_{B}\left(\Upsilon ;S\right)\right)=\lambda_{2}+\cdots +\lambda_{k+1} \end{array}$$ (16)

where λ_1_ = 0, λ_2_, …, λ_*k*+1_ are the lowest eigenvalues of L (sorted non-decreasingly), with eigenvectors corresponding to λ_2_, …, λ_*k*+1_ being the sought indicator vectors $\upsilon_{j}^{\prime }$. The clustering performed in this embedding approximates therefore the clustering Γ that minimizes NCut.

So *N*-based GSA, exploiting the normalized Laplacian L, minimizes the criterion

$$\begin{array}{c} Q^{\left[GSAL\right]}\left(\Gamma \right)=\sum_{j=1}^{k}\sum_{i\in C_{j}}\left|\right|\xi_{i}-\mu \left(C_{j}\right){\left|\right|}^{2} \end{array}$$ (17)

where ***μ***(*C*_*j*_) is the center of cluster *C*_*j*_, equal to

$$\begin{array}{c} \mu \left(C_{j}\right)=\frac{1}{|C_{j}|}\sum_{i\in C_{j}}\xi_{i} \end{array}$$ (18)

whereby ***ξ***_*i*_ = (*υ*′_1*i*_, …, *υ*′_*ki*_)^*T*^, and $\upsilon_{1}^{\prime }$ is the eigenvector of L corresponding to its eigenvalue λ_2_, $\upsilon_{2}^{\prime }$ to λ_3_ and so on.

We shall call this embedding ***ξ***_*i*_ of documents *i* from D the *N-based embedding*.

The formula (17) is in fact the target function of the *k*-means algorithm in the respective (Euclidean) embedding space. It is hence quite natural that its minimum is sought using the traditional *k*-means algorithm.

Note that if the eigenvectors of L were really the ***υ***_*j*_ indicator vectors, then *k*-means would achieve the absolute minimum equal to zero and return the intrinsic clustering and NCut optimum would be reached.

The disadvantage of L-embedding **ξ**_*i*_ is that there is no direct link between its components and the cosine similarity between textual documents from the collection D. Hence, an explanation of cluster membership is not straightforward. In the subsequent section 6 we seek a way out of this inconvenient situation. Though there is some superficial analogy to the solution in section 5, the details are making the significant difference.

## 5 Searching for clustering explanation for combinatorial Laplacian-based GSA

We face the following problem. The clustering of textual documents can be done in at least two different ways. On the one hand, we can use the traditional embedding of documents in the Term Vector Space, and then a clustering algorithm like *k*-means can be applied. On the other hand, GSA allows to embed the documents in the space spanned by eigenvector of document similarity matrix Laplacian. What is the difference?

In term-vector-space, each document is a point in a space where coordinates are terms (words) used in the document (coordinate values being e.g. term frequency-inverse document frequency). If we use *k*-means in this space, then we have a pretty simple way to explain the content of the clusters. Each cluster has a cluster center, the distance of which to the other cluster centers can be related to terms, and each document in the cluster has some similarity to the cluster center, expressed as the cosine similarity between embedding vectors, so that the cluster membership can be explained again via document terms.

The disadvantage is of course the huge dimensionality, e.g. in 1.000 documents some 10,000 terms may be used, so the Euclidean term space would be expected to be at least 10,000 dimensional.

On the other hand, we have the Graph Spectral Analysis (GSA) clustering methodology, as described in Sections 3, 4.1, and 4.2. Based on the cosine similarity between documents, we can construct a similarity matrix *S*, then its Laplacian (*L* or L), and then embed the documents into a low-dimensional space spanned by the low Laplacian eigenvectors. The number of dimensions is equal to the number *k* of clusters into which we want to cluster the data. Here also the *k*-means algorithm can be applied, but it will be much more efficient due to drastically reduced dimensionality. However, we have a problem: the coordinates in the Laplacian-induced spaces have nothing to do with the terms of the documents. How then to explain the cluster membership in terms of words from the documents?

In this section, we propose an explanation method for combinatorial Laplacian based clustering, while in Sec. 6, we present a solution for the normalized Laplacian based clustering.

It is known (Sec. 4.1) that in the discrete indicator space, clustering in the combinatorial Laplacian induced embedding produces results identical to graph clustering using RCut criterion. In the continuous space, GSA clustering method, based on the mentioned combinatorial Laplacian, approximates RCut.

Below (Sec. 5.1) we will propose a new embedding, the *K*-embedding and will demonstrate that *k*-means applied in this embedding approximately optimizes the RCut criterion (Sec. 5.2). Furthermore, we will show that *k*-means clustering in the *K*-embedding optimizes the same criterion as *k*-means in the term-vector space (Sec. 5.4). If so, then the clusters from combinatorial Laplacian embedding, from RCut clustering, from *K*-embedding and from term-vector space embedding are approximately the same. And if so, we can use the cluster explanation, originally valid for term-vector-space (Sec. 5.3), to explain cluster membership for clusters obtained via GSA method for combinatorial Laplacian.

If we take the result of any clustering method applied to textual documents, we would be able to compute for the clustering the cluster centers in Term Vector Space. We could then compute the cosine similarity between each document and the cluster center. However, this cosine similarity would tell us nothing about why the document belongs to the cluster. Our approach differs here significantly. If we take the clustering achieved in the *K*-embedding, then the cosine similarities between cluster centers in Term Vector Space will be minimized, and so these similarities justify cluster membership. This is a huge difference compared to explanation methods mentioned in [2, 34–37] and other papers.

### 5.1 A Proposal of double-centered document similarity matrix based embedding (For use with *k*-means)

Let us introduce a new embedding of the documents from D, based on [43]. Let *A* be a matrix of the form:

$$\begin{array}{c} A=11^{T}-I-S\;, \end{array}$$ (19)

where *I* is the identity matrix, and **1** is the (column) vector consisting of ones, both of appropriate dimensions. The matrix *A* is non-negative and has a diagonal equal to zero, so that it may be considered as a kind of (squared) pseudo-distance, needed by the Gower’s embedding method [44], used below. We have to assume here that the diagonal of *S* consists of zeros. Let *K* be the matrix of the (double centered) form [44]:

$$\begin{array}{c} K=-\frac{1}{2}\left(I-\frac{1}{n}11^{T}\right)A\left(I-\frac{1}{n}11^{T}\right)\;, \end{array}$$ (20)

with *n* × *n* being the dimension of *S*. **1** is an eigenvector of *K*, with the corresponding eigenvalue equal to 0. In fact, $\left(I-\frac{1}{n}11^{T}\right)1=1-\frac{1}{n}11^{T}1=1-\frac{1}{n}1n=0$. All the other eigenvectors must be orthogonal to it as *K* is real and symmetric, so for any other eigenvector **v** of *K* we have: **1**^*T*^**v** = 0.

Let Λ be the diagonal matrix of eigenvalues of *K*, and *V* the matrix where columns are corresponding (unit length) eigenvectors of *K*. Then *K* = *V*Λ*V*^*T*^. Let $z_{i}=\Lambda^{1/2}V_{i}^{T}$, where *V*_*i*_ stands for *i*-th row of *V*. Let **z**_*i*_, **z**_*ℓ*_ be the embeddings of the documents *i*, *ℓ*, resp. This embedding shall be called *K-embedding*. Then (see [43])

$$\begin{array}{c} \|z_{i}-z_{\ell }{\|}^{2}=A_{i\ell }=1-s_{i\ell } \end{array}$$ (21)

for *i* ≠ *ℓ*. Hence upon performing *k*-means clustering in this space we *de facto* try to maximize the sum of similarities within a cluster. Lingoes correction is needed, if *K* turns out to have negative eigenvalues, see [43]. The correction consists in adding 2*σ* to all elements of dissimilarity matrix *A* except for the main diagonal, which has to stay equal to zero, where *σ* ≥ −λ_*m*_ where λ_*m*_ is the smallest eigenvalue of *K*. Via adding we get a new matrix *A*′, for which we compute new *K*′ and use the prescribed embedding resulting from *K*′ and not from *K*, when performing *k*-means.

The above embedding can be used for clustering documents using the *k*-means algorithm.

Let us recall the *k*-means quality function which is minimized by *k*-means (***μ***(*C*_*j*_) = ***μ***_*j*_ is the gravity center of cluster *C*_*j*_) in the context of this new embedding.

$$\begin{array}{c} Q^{\left[Kbased\right]}\left(\Gamma \right)=\sum_{j=1}^{k}\sum_{i\in C_{j}}\left|\right|z_{i}-\mu \left(C_{j}\right){\left|\right|}^{2} \end{array}$$ (22)

(with $\mu \left(C_{j}\right)=\frac{1}{|C_{j}|}\sum_{i\in C_{j}}z_{i}$) which may be reformulated as

$$\begin{array}{c} Q^{\left[Kbased\right]}\left(\Gamma \right)=\sum_{j=1}^{k}\frac{1}{2n_{j}}\sum_{i\in C_{j}}\sum_{\ell \in C_{j}}{\left\|z_{i}-z_{\ell }\right\|}^{2}\; \end{array}$$ (23)

where *n*_*j*_ = |*C*_*j*_|. This implies

$$\begin{array}{cc} Q^{\left[Kbased\right]}\left(\Gamma \right)= & \sum_{j=1}^{k}\frac{1}{2n_{j}}\sum_{i\in C_{j}}\sum_{\begin{array}{c} \ell \in C_{j}\\ \ell \neq i \end{array}}\left(1-s_{i\ell }\right)\\ = & \sum_{j=1}^{k}\frac{1}{2n_{j}}\sum_{i\in C_{j}}\sum_{\ell \in C_{j};\ell \neq i}1-\sum_{j=1}^{k}\frac{1}{2n_{j}}\sum_{i\in C_{j}}\sum_{\ell \in C_{j};\ell \neq i}s_{i\ell }\\ = & \sum_{j=1}^{k}\frac{1}{2n_{j}}n_{j}\left(n_{j}-1\right)-\sum_{j=1}^{k}\frac{1}{2n_{j}}\sum_{i\in C_{j}}\sum_{\ell \in C_{j};\ell \neq i}s_{i\ell } \end{array}$$

that is

$$\begin{array}{c} Q^{\left[Kbased\right]}\left(\Gamma \right)=\frac{n-k}{2}-\sum_{j=1}^{k}\frac{1}{2n_{j}}\sum_{i\in C_{j}}\sum_{\ell \in C_{j};\ell \neq i}s_{i\ell }\; \end{array}$$ (24)

where *n*_*j*_ = |*C*_*j*_|, while *n*, *k* are independent of clustering.

**Fig 1:**
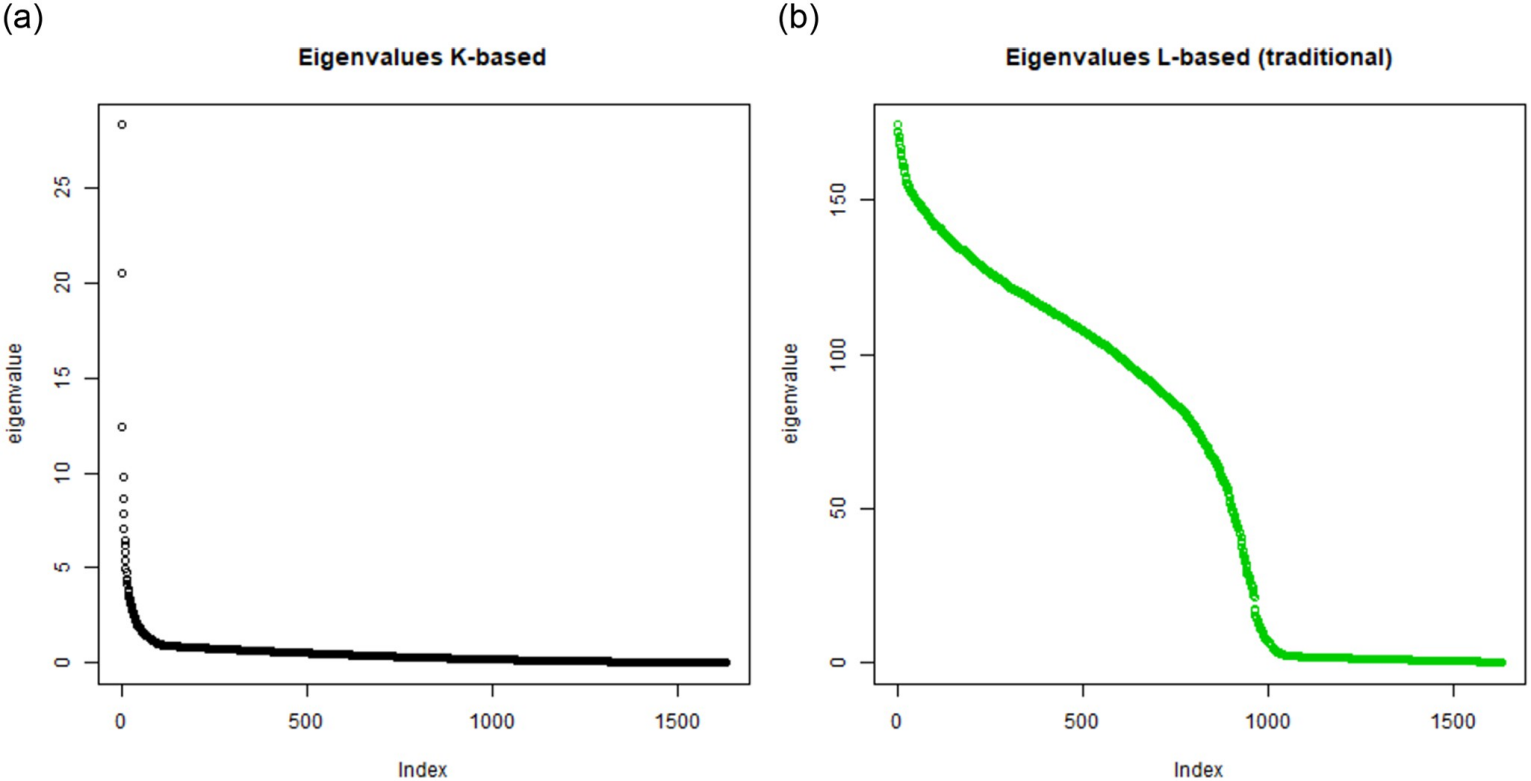
A comparison: Left—Distribution of eigenvalues under *K*-based embedding for TWT.4 data, right—Distribution of eigenvalues under *L*-based embedding for TWT.4 data.

**Fig 2:**
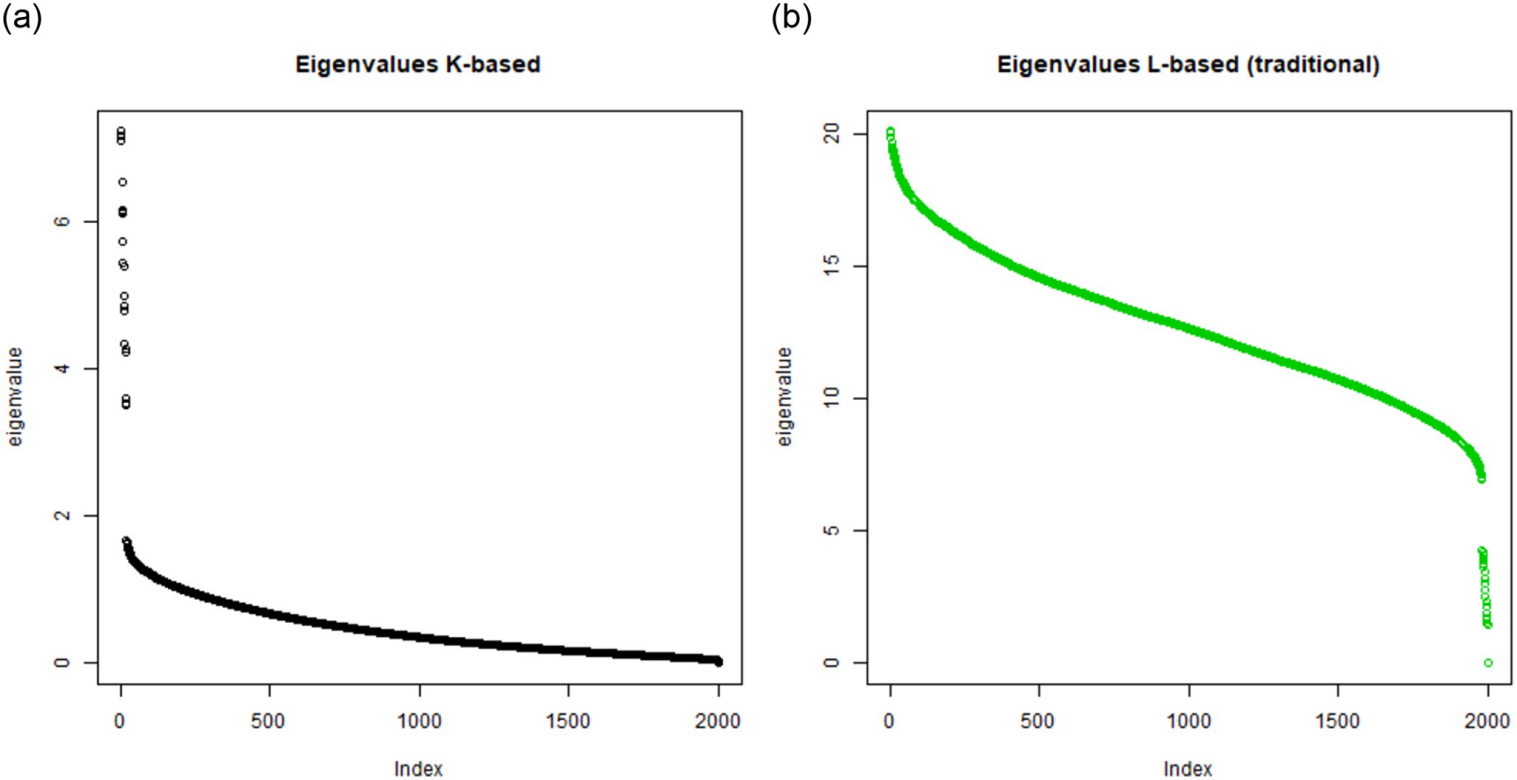
Distribution of eigenvalues under *K*-based embedding for BLK data (left) and Distribution of eigenvalues under *L*-based embedding for BLK data (right).

Instead of using all eigenvectors in representing the *K*, the top *m* eigenvalues and associated eigenvectors can be used to approximate it sufficiently. The reason is the shape of the eigenvalue spectrum as visible in Figs 1 and 2 where the leading eigenvalues are much bigger than the other ones for *K*-embedding.

### 5.2 Relationship between *L*-based clustering and *K*-based clustering

We see from Eq (24) that *k*-means applied to *K*-based embedding seeks to find the clustering that maximizes the sum of the average similarity within a cluster. On the other hand, the intention of the *L*-based GSA described previously in Sec. 4.1 is to approximate the target of RCut, which is expressed via formula (3), is to minimize the sum of average similarity to elements of other clusters. These goals are similar, but not identical, as we shall see. Let us compute the difference between them:

$$\begin{array}{cc} Q^{\left[RCut\right]}\left(\Gamma \right)-2Q^{\left[Kbased\right]}\left(\Gamma \right)= & \sum_{j=1}^{k}\frac{1}{n_{j}}\sum_{i\in C_{j}}\sum_{\ell \notin C_{j}}s_{i\ell }-\left(n-k\right)+\sum_{j=1}^{k}\frac{1}{n_{j}}\sum_{i\in C_{j}}\sum_{\ell \in C_{j};\ell \neq i}s_{i\ell }\\ = & -\left(n-k\right)+\sum_{j=1}^{k}\frac{1}{n_{j}}\sum_{i\in C_{j}}\sum_{\ell \in D}s_{i\ell }=-\left(n-k\right)+\sum_{j=1}^{k}\frac{1}{n_{j}}\sum_{i\in C_{j}}d_{ii}\\ = & -\left(n-k\right)+\sum_{j=1}^{k}\frac{V_{j}}{n_{j}} \end{array}$$

If all the clusters targeted at would be of the same size, the above expression would be a constant so that optimization of either quality function would yield the same result. Otherwise, the same results can be achieved under the following assumption: The similarities within the good clusters are above some level *g*, and those between elements of different clusters are below some level *b*. If *g*/ max_*j*_(|*C*_*j*_|) > *b*/ min_*j*_(|*C*_*j*_|), then also the optimal clustering of both is the same (if the smallest cluster is large). If these criteria are matched approximately, also the optima will be approximately the same.

### 5.3 Why is term vector space clustering explanation friendly

Let us recall the very popular method of representing documents in the so-called Term Vector Space (or Document Vector Space). One considers each document i∈D as a vector **w**_*i*_ of length equal to the number |*T*| of terms in a dictionary *T*. Each element *w*_*it*_ of the embedding vector corresponds to an indicator of the presence/absence of the term *t* in the document *i*. This indicator can be e.g. the term frequency, the inverse of the document frequency, divided by a normalising constant so that ‖**w**_*i*_‖ = 1.

The dimensionality of this embedding is huge compared to those based on *L* or *K*, but it has some nice properties. First of all, it defines document similarities in that $s_{i\ell }=w_{i}^{T}w_{\ell }$ by definition. Consider the *k*-mean clustering in such a space. The following criterion can be minimised:

$$\begin{array}{c} Q^{\left[TVS\right]}\left(\Gamma \right)=\sum_{j=1}^{k}\sum_{i\in C_{j}}\left|\right|w_{i}-\mu \left(C_{j}\right){\left|\right|}^{2} \end{array}$$ (25)

(with $\mu \left(C_{j}\right)=\frac{1}{|C_{j}|}\sum_{i\in C_{j}}w_{i}$) which may be reformulated as

$$\begin{array}{c} Q^{\left[TVS\right]}\left(\Gamma \right)=\sum_{j=1}^{k}\frac{1}{2n_{j}}\sum_{i\in C_{j}}\sum_{\ell \in C_{j}}{\left\|w_{i}-w_{\ell }\right\|}^{2}\; \end{array}$$ (26)

where *n*_*j*_ is the cardinality of *C*_*j*_.

Obviously, for *i* ≠ *ℓ*

$$\|w_{i}-w_{\ell }{\|}^{2}=\|w_{i}{\|}^{2}-2w_{i}^{T}w_{\ell }+{\left\|w_{\ell }\right\|}^{2}=2-2s_{i\ell }$$

This means that

$$\begin{array}{c} Q^{\left[TVS\right]}\left(\Gamma \right)=\sum_{j=1}^{k}\frac{1}{n_{j}}\sum_{i\in C_{j}}\sum_{\ell \in C_{j};\ell \neq i}\left(1-s_{i\ell }\right) \end{array}$$ (27)

$$\begin{array}{cc} = & \sum_{j=1}^{k}\frac{1}{n_{j}}\sum_{i\in C_{j}}\sum_{\ell \in C_{j};\ell \neq i}1-\sum_{j=1}^{k}\frac{1}{n_{j}}\sum_{i\in C_{j}}\sum_{\ell \in C_{j};\ell \neq i}s_{i\ell } \end{array}$$ (28)

$$\begin{array}{cc} = & \sum_{j=1}^{k}\frac{1}{n_{j}}\left(n_{j}-1\right)n_{j}-\sum_{j=1}^{k}\frac{1}{n_{j}}\sum_{i\in C_{j}}\sum_{\ell \in C_{j};\ell \neq i}s_{i\ell } \end{array}$$ (29)

$$\begin{array}{cc} = & n-k-\sum_{j=1}^{k}\frac{1}{n_{j}}\sum_{i\in C_{j}}\sum_{\ell \in C_{j};\ell \neq i}s_{i\ell } \end{array}$$ (30)

If a clustering was performed via *k*-means in this embedding and cluster set Γ was obtained, then we have the following possibility to explain each cluster *C*_*j*_ by the characteristic terms: Compute cluster centers ***μ***_*j*_ = ***μ***(*C*_*j*_). Let denote *μ*_*jt*_ the element of ***μ***_*j*_ related to term *t*. Sort terms from *T* into a sequence *t*_1_, …, *t*_|*T*|_ so that $\mu_{jt_{p}}\geq \mu_{jt_{p+1}}$. Then take the leading *m* terms *t*_1_, …, *t*_*m*_ as the explanation of the cluster.

Let us illustrate this method of cluster explanation with the following simple example. We considered clustering of tweets related to two hashtags (see the experimental section for details): #anjisalvacion and #puredoctrinesofchrist. We clustered the data using *K*-based method, described in Sec. 5.1, that is equivalent to clustering in the Term Vector Space, as shown in Sec. 5.4, and got two clusters characterized by the following 10 top words (according to the above prescription):

- Cluster 1: “anjisalvacion”, “anji”, “god”, “dalampasigan”, “esv”, “life”, “people”, “happy”, “love”, “day”
- Cluster 2: “king”, “proverbs”, “god”, “luke”, “lord”, “psalm”, “christ”, “man”, “jesus”, “hath”

For comparison see the top words in the respective hashtags:

- hashtag set #anjisalvacion: “anjisalvacion”, “anji”, “dalampasigan”, “life”, “happy”, “feelstheconcert”, “birthday”, “people”, “salvacion”, “mv”
- hashtag set #puredoctrinesofchrist: “king”, “god”, “proverbs”, “esv”, “lord”, “christ”, “eli”, “let”, “jesus”, “man”

One can see that the term-vector-space based explanation looks quite reasonable.

You may also explain the cluster membership of a document in a similar way. The document similarity of a document *i* to its cluster center is given as

$$\begin{array}{c} w_{i}^{T}\mu_{j}=\sum_{t\in T}w_{it}\mu_{jt} \end{array}$$ (31)

Sort terms from *T* into a sequence *t*_1_, …, *t*_|*T*|_ so that $w_{it_{p}}\mu_{jt_{p}}\geq w_{it_{p+1}}\mu_{jt_{p+1}}$. Then take the leading *m* terms *t*_1_, …, *t*_*m*_ as the explanation of the membership of the document *i* in cluster *C*_*j*_.

But the aforementioned explanation of clusters via the terms does not take into account the distinction from the other clusters.

Below we make a new proposal of relative cluster explanation. For the cluster *j* consider the following expression

$$\begin{array}{c} ClDiff\left(j\right)=\sum_{j^{\prime }=1}^{k}{\left\|\mu_{j}-\mu_{j^{\prime }}\right\|}^{2} \end{array}$$ (32)

which is the sum of squared differences between the given cluster and the other ones. It can be rewritten as

$$\begin{array}{c} ClDiff\left(j\right)=\sum_{j^{\prime }=1}^{k}\sum_{t\in T}{\left(\mu_{jt}-\mu_{j^{\prime }t}\right)}^{2}=\sum_{t\in T}(\sum_{j^{\prime }=1}^{k}{\left(\mu_{jt}-\mu_{j^{\prime }t}\right)}^{2}) \end{array}$$ (33)

Based on this, just sort the terms from *T* into a sequence *t*_1_, …, *t*_|*T*|_ so that for each *t*_*p*_ in this sequence $\left(\sum_{j^{\prime }=1}^{k}{\left(\mu_{jt_{p}}-\mu_{j^{\prime }t_{p}}\right)}^{2})\geq (\sum_{j^{\prime }=1}^{k}{\left(\mu_{jt_{p+1}}-\mu_{j^{\prime }t_{p+1}}\right)}^{2}\right)$. Then take the leading *m* terms *t*_1_, …, *t*_*m*_ as the first step explanation of the cluster. However, further elaboration is needed because the distinction between two clusters may result from the fact that the term is present in one, but absent in the other (which would be quite simple to detect), or has a higher or lower tfidf value at this position, which is not that easy to decide because there is a multitude of clusters. Therefore, if term *t*_*p*_ is important, consider the expression $\left(\sum_{j^{\prime }=1}^{k}{\left(\mu_{jt_{p}}-\mu_{j^{\prime }t_{p}}\right)}^{2}\right)$ and compute its derivative on $\mu_{jt_{p}}$ that is

$$\begin{array}{c} \frac{d}{d\mu_{jt_{p}}}(\sum_{j^{\prime }=1}^{k}{\left(\mu_{jt_{p}}-\mu_{j^{\prime }t_{p}}\right)}^{2})=\sum_{j^{\prime }=1}^{k}2\left(\mu_{jt_{p}}-\mu_{j^{\prime }t_{p}}\right) \end{array}$$ (34)

If this derivative is positive at $\mu_{jt_{p}}$, then the presence of the term *t*_*p*_ is important for cluster distinction, otherwise its absence.

This would lead to the following cluster description.

- Cluster 1: “(++) anjisalvacion” “(++) anji” “(-) king” “(++) dalampasigan” “(++) esv” “(++) life” “(++) god” “(++) happy” “(++) people” “(++) love”
- Cluster 2: “(-) anjisalvacion” “(-) anji” “(++) king” “(-) dalampasigan” “(-) esv” “(-) life” “(-) god” “(-) happy” “(-) people” “(-) love”

Terms marked with (++) are those the presence of which is in favour of the membership in the cluster, while (-) is discouraging membership. So for example the term “anjisalvacion” is more likely to appear in texts of the first cluster than in those of the second.

### 5.4 Equivalence between *K*-based clustering and term/document vector based clustering

A quick look at formulas (24) and (30) reveals that both clustering criteria are identical. Therefore clustering in *K*-based embedding and clustering in Term Vector Space optimize the same target function. What is the difference? The *K*-embedding is lower dimensional, as the length of eigenvectors equals the number of documents. Here, the number of dimensions is equal to the richness of the vocabulary, which may be 10 times as high or more. Hence the clustering under *K*-embedding will be significantly faster.

What do we gain then via Term Vector Space embedding? We have already seen that under-balanced clusters clustering in *K*-based embedding approximates RCut clustering which on the other hand is approximated by *L*-based clustering. Therefore, the application of cluster and cluster membership explanation methods outlined in Sec. 5.3 to the results of *L*-based spectral clustering method is justified.

In summary, we have pointed out in this section that the traditional *L*-embedding lost the direct relation between datapoint distances and the cosine similarity of documents. This is a serious disadvantage because *k*-means is applied in GSA clusters based on distances in embedding space, not similarities between documents. We have shown that there exists a *K* embedding having approximately the same general goal as *L*-embedding (see Sec. 5.2), but with the property that distances in the space are directly translated to similarities so that *k*-means applied in this embedding optimizes on the similarities within a cluster directly. In the third embedding, the Term Vector Space embedding, the similarities can be computed directly as cosine similarity or based on Euclidean distances. This duality allows for precise pointing at sources of similarities of the cluster elements and at sources of dissimilarities in terms of words of the documents.

In this way the problem of GSA explanation is overcome in that membership reason can be given in terms of sets of decisive words.

## 6 Searching for clustering explanation for normalized Laplacian-based GSA

The currently more popular normalized Laplacian based spectral clustering (see Eq (2)) constitutes a bigger challenge for explanation as the translation to cosine similarity is not that straightforward. Note that the issues we are interested in were discussed by [45].

It is known (Section 4.2) that in the discrete indicator space, clustering in the normalised Laplacian induced embedding gives identical results to graph clustering using the NCut criterion. But in the continuous space, GSA based on the mentioned normalised Laplacian only approximates NCut. To bridge the gap between Term Vector Space and NCuts, we will proceed similarly to the treatment of RCut in Section 5.1. So first we propose a new embedding, the M embedding (Section 6.1), and will show that the *weighted* *k*-means used in this embedding exactly optimizes the NCut criterion (Section 6.2). When we talk about weighted *k*-means, we mean that the items are weighted, but not the features. We also assume that the weights are given in advance and are not changed during the execution of the weighted *k*-means algorithm. Note that previously (for *K*-embedding), we used the plain *k*-means algorithm.

We will show that weighted *k*-means clustering in the M-embedding optimizes the *same* criterion as weighted *k*-means in the term-vector space. (In the case of RCut, only a *similar* criterion was optimized). If so, then the clusters from normalized Laplacian embedding and from NCut clustering are approximately the same (see Sec. 4.2), while those from NCut, from M-embedding and from term-vector space embedding are identical. And if so, we can use the cluster explanation, originally valid for term-vector space, to explain cluster membership for clusters obtained by the GSA method for normalized Laplacian, but with the correction that the explanation takes into account the document weights.

### 6.1 A proposal of double-centered “normalized” document similarity matrix based embedding (For use with weighted *k*-means)

We suggest to use the A matrix of the following form. E be a matrix of the following form

$$\begin{array}{c} E=11^{T}-I\;. \end{array}$$ (35)

Then define

$$\begin{array}{c} A=D^{-1}\left(ED+DE-2S\right)D^{-1}\;. \end{array}$$ (36)

with *D*, *S* being defined as previously. Let M be the matrix of the form:

$$\begin{array}{c} M=-\frac{1}{2}\left(I-\frac{1}{n}11^{T}\right)A\left(I-\frac{1}{n}11^{T}\right)\;. \end{array}$$ (37)

We proceed with M in a similar way as with *K* matrix. Note that **1** is an eigenvector of M, with the corresponding eigenvalue equal to 0. All the other eigenvectors must be orthogonal to it as M is real and symmetric, so for any other eigenvector **v** of M we have: **1**^*T*^**v** = 0.

Let Λ be the diagonal matrix of eigenvalues of M, and *V* the matrix where columns are corresponding (unit length) eigenvectors of M. Then $M=V\Lambda V^{T}$. Let $\zeta_{i}=\Lambda^{1/2}V_{i}^{T}$, where *V*_*i*_ stands for *i*-th row of *V*. Let ***ζ***_*i*_, ***ζ***_*ℓ*_ be the embeddings of the documents *i*, *ℓ*, resp. This embedding shall be called M-*embedding*. Then

$$\begin{array}{c} \|\zeta_{i}-\zeta_{\ell }{\|}^{2}=A_{i\ell }=\left(d_{ii}+d_{\ell \ell }-2s_{i\ell }\right)/\left(d_{ii}d_{\ell \ell }\right) \end{array}$$ (38)

for *i* ≠ *ℓ*, and zero otherwise. Let us now discuss performing weighted *k*-means clustering on the vectors ***ζ***_*i*_ with weights amounting to *d*_*ii*_ respectively.

Let us use the following weighting of documents: *ω*_*i*_ = *d*_*ii*_. Clustering via weighted *k*-means with weights *ω*_*i*_ in the M embedding will optimize the following criterion

$$\begin{array}{c} Q^{\left[Mbased\right]}\left(\Gamma ;\omega \right)=\sum_{j=1}^{k}\sum_{i\in C_{j}}\omega_{i}{\left\|\zeta_{i}-\mu_{\omega }\left(C_{j}\right)\right\|}^{2} \end{array}$$ (39)

whereby

$$\mu_{\omega }\left(C_{j}\right)=\frac{\sum_{\in C_{j}}\omega_{i}\zeta_{i}}{\sum_{\in C_{j}}\omega_{i}}=\frac{1}{V_{j}}\sum_{\in C_{j}}\omega_{i}\zeta_{i}$$

which may be reformulated as

$$\begin{array}{c} Q^{\left[Mbased\right]}\left(\Gamma ;\omega \right)=\sum_{i=1}^{n}\sum_{j=1}^{k}u_{ij}\omega_{i}\|\zeta_{i}-\mu_{\omega }{j\|}^{2}=\sum_{j=1}^{k}\frac{1}{V_{j}}\sum_{\zeta_{i},\zeta_{\ell }\in C_{j}}\omega_{i}\omega_{\ell }{\left\|\zeta_{i}-\zeta_{\ell }\right\|}^{2} \end{array}$$ (40)

$$\begin{array}{cc} & =\sum_{j=1}^{k}\frac{1}{2V_{j}}\sum_{i\in C_{j}}\sum_{\ell \in C_{j}}\omega_{i}\omega_{\ell }{\left\|\zeta_{i}-\zeta_{\ell }\right\|}^{2}\;\; \end{array}$$ (41)

$$\begin{array}{cc} & =\sum_{j=1}^{k}\frac{1}{2V_{j}}\sum_{i\in C_{j}}\sum_{\begin{array}{c} \ell \in C_{j}\\ \ell \neq i \end{array}}\left(d_{ii}+d_{\ell \ell }-2s_{i\ell }\right)\;\; \end{array}$$ (42)

$$\begin{array}{cc} & =\sum_{j=1}^{k}\frac{1}{2V_{j}}((\sum_{i\in C_{j}}\sum_{\begin{array}{c} \ell \in C_{j}\\ \ell \neq i \end{array}}d_{ii})+(\sum_{i\in C_{j}}\sum_{\begin{array}{c} \ell \in C_{j}\\ \ell \neq i \end{array}}d_{\ell \ell })-(\sum_{i\in C_{j}}\sum_{\begin{array}{c} \ell \in C_{j}\\ \ell \neq i \end{array}}2s_{i\ell }))\; \end{array}$$ (43)

$$\begin{array}{cc} & =\sum_{j=1}^{k}\frac{1}{2V_{j}}(\left(\right|C_{j}\left|-1\right)V_{j}+\sum_{i\in C_{j}}(V_{j}-d_{ii})-(\sum_{i\in C_{j}}\sum_{\begin{array}{c} \ell \in C_{j}\\ \ell \neq i \end{array}}2s_{i\ell }))\; \end{array}$$ (44)

$$\begin{array}{cc} & =\sum_{j=1}^{k}\frac{1}{2V_{j}}(\left(\right|C_{j}\left|-1\right)V_{j}+(\sum_{i\in C_{j}}V_{j}-\sum_{i\in C_{j}}d_{ii})-(\sum_{i\in C_{j}}\sum_{\begin{array}{c} \ell \in C_{j}\\ \ell \neq i \end{array}}2s_{i\ell }))\;\; \end{array}$$ (45)

$$\begin{array}{cc} & =\sum_{j=1}^{k}\frac{1}{2V_{j}}(\left(\right|C_{j}\left|-1\right)V_{j}+(|C_{j}|V_{j}-V_{j})-(\sum_{i\in C_{j}}\sum_{\begin{array}{c} \ell \in C_{j}\\ \ell \neq i \end{array}}2s_{i\ell }))\; \end{array}$$ (46)

That is

$$\begin{array}{c} Q^{\left[Mbased\right]}\left(\Gamma ;\omega \right)=\sum_{j=1}^{k}\frac{1}{2V_{j}}(2(|C_{j}\left|-1\right)V_{j}-(\sum_{i\in C_{j}}\sum_{\begin{array}{c} \ell \in C_{j}\\ \ell \neq i \end{array}}2s_{i\ell }))\; \end{array}$$ (47)

$$\begin{array}{cc} & =\sum_{j=1}^{k}\frac{1}{2V_{j}}(2(|C_{j}\left|-2\right)V_{j}+2(V_{j}-\sum_{i\in C_{j}}\sum_{\begin{array}{c} \ell \in C_{j}\\ \ell \neq i \end{array}}s_{i\ell }))\; \end{array}$$ (48)

$$\begin{array}{cc} & =\sum_{j=1}^{k}\frac{1}{2V_{j}}(2(|C_{j}\left|-2\right)V_{j}+2(\sum_{i\in C_{j}}d_{ii}-\sum_{i\in C_{j}}\sum_{\begin{array}{c} \ell \in C_{j}\\ \ell \neq i \end{array}}s_{i\ell }))\; \end{array}$$ (49)

$$\begin{array}{cc} & =\sum_{j=1}^{k}\frac{1}{2V_{j}}(2(|C_{j}\left|-2\right)V_{j}+2\sum_{i\in C_{j}}(d_{ii}-\sum_{\begin{array}{c} \ell \in C_{j}\\ \ell \neq i \end{array}}s_{i\ell }))\; \end{array}$$ (50)

$$\begin{array}{cc} & =\sum_{j=1}^{k}\frac{1}{2V_{j}}(2(|C_{j}\left|-2\right)V_{j}+2\sum_{i\in C_{j}}(\sum_{\ell \notin C_{j}}s_{i\ell }))\; \end{array}$$ (51)

$$\begin{array}{cc} & =\sum_{j=1}^{k}\frac{1}{V_{j}}\left(\right|C_{j}\left|-2\right)V_{j}+\sum_{j=1}^{k}\frac{1}{V_{j}}(\sum_{i\in C_{j}}(\sum_{\ell \notin C_{j}}s_{i\ell }))\;\; \end{array}$$ (52)

$$\begin{array}{cc} & =n-2k+\sum_{j=1}^{k}\frac{1}{V_{j}}\sum_{i\in C_{j}}\sum_{\ell \notin C_{j}}s_{i\ell }\; \end{array}$$ (53)

(see (11) for comparison).

### 6.2 Relationship between *N*-based clustering and M-based clustering

From Eqs (11) and (53) we see immediately that

$$\begin{array}{c} Q^{\left[Mbased\right]}\left(\Gamma ;\omega \right)=n-2k+Q^{\left[NCut\right]}\left(\Gamma \right) \end{array}$$ (54)

As *n* − 2*k* is a constant, minimizing one criterion minimizes the second one. As *N*-based Clustering (clustering using the normalized Laplacian L) has the same target as NCut clustering, we see that we have here a better situation than for *K*-embedding versus *L*-based clustering. Lingoes correction is needed, if M turns out to have negative eigenvalues, see [43] and the earlier remark on this correction.

Note that a similar topic was handled by [45], with an extension by [46]. However, they sought equivalence between spectral approximation to NCut and weighted *k*-means clustering, while we looked for equivalence directly to NCut clustering. We stress also the capability to go over to cluster explanation.

### 6.3 How to make weighted term vector space clustering explanation friendly

In order to parallel the dissimilarity measure from formula (38), the squared dissimilarity between documents *i* and *ℓ* would have to be defined in a specific way. Consider the **w**_*i*_ vectors representing embedding in Term Vector Space that was introduced in Section 5.3. Consider a modified vector in the Term Vector Space:

$$\begin{array}{c} w_{i}^{\prime }=(\frac{w_{i}}{\omega_{i}},g_{i}) \end{array}$$ (55)

where **g**_**i**_ is a vector of dimension *n*, equal to zero everywhere except the *i*th element, $g_{ii}=\frac{\sqrt{\omega_{i}-1}}{\omega_{i}}$. With this notation, let us compute the squared distance between two documents: (for different *i*, *ℓ*)

$$\begin{array}{cc} \|w_{i}^{\prime }-w_{\ell }^{\prime }{\|}^{2} & =\|w_{i}^{\prime }{\|}^{2}+{\left\|w_{\ell }^{\prime }\right\|}^{2}-2w_{\ell }^{\prime }Tw_{i}^{\prime } \end{array}$$ (56)

$$\begin{array}{cc} & =\frac{\omega_{i}-1+1}{\omega_{i}^{2}}+\frac{\omega_{\ell }-1+1}{\omega_{\ell }^{2}}-2\frac{s_{i\ell }}{\omega_{i}\omega_{\ell }}=\frac{1}{\omega_{i}}+\frac{1}{\omega_{\ell }}-2\frac{s_{i\ell }}{\omega_{i}\omega_{\ell }} \end{array}$$ (57)

The dissimilarity is greater when the vectors are longer, but smaller if the dot product is bigger. Under this assumption, one has to perform weighted *k*-means clustering (for weighted kernel-*k*-means see e.g. [45]):

$$\begin{array}{c} Q^{\left[\omega TVS\right]}\left(\Gamma ;\omega \right)=\sum_{j=1}^{k}\frac{1}{2V_{j}}\sum_{i\in C_{j}}\sum_{\begin{array}{c} \ell \in C_{j}\\ \ell \neq i \end{array}}\omega_{i}\omega_{\ell }{\left\|w_{i}^{\prime }-w_{\ell }^{\prime }\right\|}^{2} \end{array}$$ (58)

$$\begin{array}{cc} & =\sum_{j=1}^{k}\frac{1}{2V_{j}}\sum_{i\in C_{j}}\sum_{\begin{array}{c} \ell \in C_{j}\\ \ell \neq i \end{array}}\omega_{i}\omega_{\ell }(\frac{1}{\omega_{i}}+\frac{1}{\omega_{\ell }}-2\frac{s_{i\ell }}{\omega_{i}\omega_{\ell }}) \end{array}$$ (59)

$$\begin{array}{cc} & =\sum_{j=1}^{k}\frac{1}{2V_{j}}\sum_{i\in C_{j}}\sum_{\begin{array}{c} \ell \in C_{j}\\ \ell \neq i \end{array}}(\omega_{i}+\omega_{\ell }-2s_{i\ell }) \end{array}$$ (60)

which is identical with Eq (42) showing equivalence with $Q^{\left[Mbased\right]}\left(\Gamma ;\omega \right)$.

Note that the **w**′_*i*_ vectors are of higher dimension than those used in the kernel approach in the M. There is no need to use them in practice during the clustering process. One uses them only “mentally” for the sake of explanation. As proven in Section 6.4, clusters resulting from clustering the **w**′_*i*_ vectors are the same as clusters resulting from clustering in the M-embedding.

The advantage of this embedding is that we can use it to look for best characterizing terms as in the case of term space embedding described in Section 5.3.

This means: If a clustering was performed via weighted *k*-means in this embedding and cluster set Γ was obtained, then we have the following possibility to explain each cluster *C*_*j*_ by the characteristic terms: Compute cluster centers $\mu_{j}=\mu \left(C_{j}\right)=\frac{\sum_{i\in C_{j}}\omega_{i}w_{i}^{\prime }}{\sum_{i\in C_{j}}\omega_{i}}=\frac{\sum_{i\in C_{j}}\left(w_{i},\omega_{i}g_{i}\right)}{\sum_{i\in C_{j}}\omega_{i}}$. Let denote *μ*_*jt*_ the element of ***μ***_*j*_ related to term *t*. Sort terms from *T* into a sequence *t*_1_, …, *t*_|*T*|_ so that $\mu_{jt_{p}}\geq \mu_{jt_{p+1}}$. Then take the leading *m* terms *t*_1_, …, *t*_*m*_ as the explanation of the cluster.

Let us illustrate this method of cluster explanation with the following simple example. We considered clustering of tweets related to two hashtags (see the experimental section for details): #anjisalvacion and #puredoctrinesofchrist. We clustered the data using M-based method, described below, that is equivalent to clustering in the Term Vector Space, and got two clusters characterized by the following 10 top words (according to the above prescription):

- Cluster 1: “anjisalvacion”, “anji” “king”, “god”, “dalampasigan”, “life”, “people”, “proverbs”, “happy” “feelstheconcert”
- Cluster 2: “esv”, “god”, “proverbs”, “king”, “eli”, “lord”, “christ”, “words”, “spirit”, “holy”

Formulas from Section 5.3 for explaining cluster membership (31) and for explaining a cluster against other clusters (32) can be applied analogously.

### 6.4 Equivalence between M-based clustering and weighted term/document vector based clustering

Essentially, we have shown the equivalence in the previous section 6.3 (compare formulas (42) and (60)). There is, however, one weakness of the approach: $g_{i}=\frac{\sqrt{\omega_{i}-1}}{\omega_{i}}$ may be a complex number if *ω*_*i*_ is too small. We can get around the problem if *ω*_*i*_ is not equal to *d*_*ii*_, but rather to a multiplicity of it, that is *ω*_*i*_ = *f* ⋅ *d*_*ii*_. In such a case $V_{j}$ needs to be redefined for the purpose of the weighted Term/Document Vector embedding as sum of *ω*_*i*_

$$V_{j}^{*}=\sum_{i\in C_{j}}\omega_{i}$$

In such a case the cost function will not be identical with but rather in a linear relation with Eq (42). This reformulation will go as follows:

$$\begin{array}{c} Q^{\left[\omega TVS\right]}\left(\Gamma ;\omega \right)=\sum_{j=1}^{k}\frac{1}{2V_{j}^{*}}\sum_{i\in C_{j}}\sum_{\begin{array}{c} \ell \in C_{j}\\ \ell \neq i \end{array}}\omega_{i}\omega_{\ell }{\left\|w_{i}^{\prime }-w_{\ell }^{\prime }\right\|}^{2} \end{array}$$ (61)

$$\begin{array}{cc} & =\sum_{j=1}^{k}\frac{1}{2V_{j}^{*}}\sum_{i\in C_{j}}\sum_{\begin{array}{c} \ell \in C_{j}\\ \ell \neq i \end{array}}\omega_{i}\omega_{\ell }(\frac{1}{\omega_{i}}+\frac{1}{\omega_{\ell }}-2\frac{s_{i\ell }}{\omega_{i}\omega_{\ell }}) \end{array}$$ (62)

$$\begin{array}{cc} & =\sum_{j=1}^{k}\frac{1}{2V_{j}^{*}}\sum_{i\in C_{j}}\sum_{\begin{array}{c} \ell \in C_{j}\\ \ell \neq i \end{array}}(\omega_{i}+\omega_{\ell }-2s_{i\ell })\; \end{array}$$ (63)

$$\begin{array}{cc} & =\sum_{j=1}^{k}\frac{1}{2V_{j}^{*}}((\sum_{i\in C_{j}}\sum_{\begin{array}{c} \ell \in C_{j}\\ \ell \neq i \end{array}}\omega_{i})+(\sum_{i\in C_{j}}\sum_{\begin{array}{c} \ell \in C_{j}\\ \ell \neq i \end{array}}\omega_{\ell })-(\sum_{i\in C_{j}}\sum_{\begin{array}{c} \ell \in C_{j}\\ \ell \neq i \end{array}}2s_{i\ell }))\; \end{array}$$ (64)

$$\begin{array}{cc} & =\sum_{j=1}^{k}\frac{1}{2V_{j}^{*}}(\left(\right|C_{j}\left|-1\right)V_{j}^{*}+\sum_{i\in C_{j}}(V_{j}^{*}-\omega_{i})-(\sum_{i\in C_{j}}\sum_{\begin{array}{c} \ell \in C_{j}\\ \ell \neq i \end{array}}2s_{i\ell }))\; \end{array}$$ (65)

$$\begin{array}{cc} & =\sum_{j=1}^{k}\frac{1}{2V_{j}^{*}}(\left(\right|C_{j}\left|-1\right)V_{j}^{*}+(\sum_{i\in C_{j}}V_{j}^{*}-\sum_{i\in C_{j}}\omega_{i})-(\sum_{i\in C_{j}}\sum_{\begin{array}{c} \ell \in C_{j}\\ \ell \neq i \end{array}}2s_{i\ell }))\;\; \end{array}$$ (66)

$$\begin{array}{cc} & =\sum_{j=1}^{k}\frac{1}{2V_{j}^{*}}(\left(\right|C_{j}\left|-1\right)V_{j}^{*}+(|C_{j}|V_{j}^{*}-V_{j}^{*})-(\sum_{i\in C_{j}}\sum_{\begin{array}{c} \ell \in C_{j}\\ \ell \neq i \end{array}}2s_{i\ell }))\; \end{array}$$ (67)

That is

$$\begin{array}{c} Q^{\left[\omega TVS\right]}\left(\Gamma ;\omega \right)=\sum_{j=1}^{k}\frac{1}{2V_{j}^{*}}(2(|C_{j}\left|-1\right)V_{j}^{*}-(\sum_{i\in C_{j}}\sum_{\begin{array}{c} \ell \in C_{j}\\ \ell \neq i \end{array}}2s_{i\ell }))\; \end{array}$$ (68)

$$\begin{array}{cc} & =\sum_{j=1}^{k}\frac{1}{2V_{j}^{*}}(2(|C_{j}\left|-1-\frac{1}{f}\right)V_{j}^{*}+2(\frac{V_{j}^{*}}{f}-\sum_{i\in C_{j}}\sum_{\begin{array}{c} \ell \in C_{j}\\ \ell \neq i \end{array}}s_{i\ell }))\; \end{array}$$ (69)

$$\begin{array}{cc} & =\sum_{j=1}^{k}\frac{1}{2V_{j}^{*}}(2(|C_{j}\left|-1-\frac{1}{f}\right)V_{j}^{*}+2(\sum_{i\in C_{j}}d_{ii}-\sum_{i\in C_{j}}\sum_{\begin{array}{c} \ell \in C_{j}\\ \ell \neq i \end{array}}s_{i\ell }))\; \end{array}$$ (70)

$$\begin{array}{cc} & =\sum_{j=1}^{k}\frac{1}{2V_{j}^{*}}(2(|C_{j}\left|-1-\frac{1}{f}\right)V_{j}^{*}+2\sum_{i\in C_{j}}(d_{ii}-\sum_{\begin{array}{c} \ell \in C_{j}\\ \ell \neq i \end{array}}s_{i\ell }))\; \end{array}$$ (71)

$$\begin{array}{cc} & =\sum_{j=1}^{k}\frac{1}{2V_{j}^{*}}(2(|C_{j}\left|-1-\frac{1}{f}\right)V_{j}^{*}+2\sum_{i\in C_{j}}(\sum_{\ell \notin C_{j}}s_{i\ell }))\; \end{array}$$ (72)

$$\begin{array}{cc} & =\sum_{j=1}^{k}\frac{1}{V_{j}^{*}}\left(\right|C_{j}\left|-1-\frac{1}{f}\right)V_{j}^{*}+\sum_{j=1}^{k}\frac{1}{V_{j}^{*}}(\sum_{i\in C_{j}}(\sum_{\ell \notin C_{j}}s_{i\ell }))\;\; \end{array}$$ (73)

$$\begin{array}{cc} & =n-\left(1+\frac{1}{f}\right)k+\sum_{j=1}^{k}\frac{1}{fV_{j}}\sum_{i\in C_{j}}\sum_{\ell \notin C_{j}}s_{i\ell }\; \end{array}$$ (74)

That is

$$f(Q^{\left[\omega TVS\right]}\left(\Gamma ;\omega \right)-n-\left(1+\frac{1}{f}\right)k)=\sum_{j=1}^{k}\frac{1}{fV_{j}}\sum_{i\in C_{j}}\sum_{\ell \notin C_{j}}s_{i\ell }=Q^{\left[Mbased\right]}\left(\Gamma ;\omega \right)-n+2k$$

As already mentioned, there exist no “standard” method of explaining the contents of clusters obtained using the *N*-embedding of Normalized Laplacian Based Graph Spectral Clustering method. The “universal” methods presented in Section 2 do not give a warranty that the distance between the cluster center and the given document has anything to do with optimization method underlying the clustering methodology. Our approach to GSA based on normalized Laplacian is different and even more advantageous than for combinatorial Laplacians. It goes along the following line: NCut clustering, among others applied to text document collections, is equivalent to clustering using *N*-embedding as generally known (see Section 4.2), that is the classical Normalized Laplacian based Graph Spectral Clustering under some rigid assumptions on the form of cluster membership indicator vectors. Upon relaxation of these assumptions, *N*-embedding based clustering approximates NCut clustering. But the normalized Laplacian matrix eigenvectors have no meaningful interpretation in terms of document contents. So we proposed an M-embedding based clustering method (Section 6.1) that has a target equivalent to NCut (Section 6.2). Note that this is different from *K*-embedding which has only approximately equivalent clustering target as RCut. However, the M-embedding vectors have also no direct relationship to text document terms. Therefore, we investigated the weighted Term Vector Space (wTVS) embedding vectors the components of which have a direct correspondence to terms in textual documents, Section 6.3. The target of wTVS-embedding is the same of M-embedding (Section 6.4) which is the same as of NCut (Section 6.2) which is approximated by *N*-embedding (Section 4.2). So as clusters in wTVS-embedding can be explained in terms of weighted Term Vector Space (that is the leading terms of cluster center representation, and of each document representation, as explained already at the end of Section 5.3), so same explanation holds for M-embedding, NCUT and *N*-embedding.

## 7 Experiments

Through experiments,

- we demonstrate, by inspection of their eigenvalue spectrogram, that *K* matrix is unrelated to *L* matrix;
- we demonstrate that *L*-based embedding differs from *K*-based embedding in that *L*-based embedding is poorly related to the similarity measures, while *K*-based embedding is correlated with similarity;
- finally, we demonstrate that both are similarly well suited for text clustering in that we show that they restore groups of tweets sharing the same hashtag with similar performance.

### 7.1 Data

In the experiments, we use three sets of data:

- A synthetic dataset BLK of 2000 “product descriptions” divided into 4 classes, hence referred to as BLK4); the dataset was generated by a random generator providing random descriptive texts, but characterized by a clear block-structure relationships within the classes (generator was the BLK_read.R program, in the directory R of https://github.com/ipipan-barstar/PLOS.EGSCoTD).
- The set TWT.4, is a collection of tweets related to hashtags #anjisalvacion, #lolinginlove, #nowplaying and #puredoctrinesofchrist from TWT.10 dataset.
- The set TWT.10, being a collection of tweets related to hashtags listed in Table 2 (available in directory Data of https://github.com/ipipan-barstar/PLOS.EGSCoTD). Only tweets were selected in which only one hashtag appeared (only once). The processing code was placed in the directory Python at the same WWW address.

**Table 2:** TWT.10 dataset—Hashtags and cardinalities of the set of related tweets used in the experiments.

No.	hashtag	count
0	90dayfiance	316
1	tejran	345
2	ukraine	352
3	tejasswiprakash	372
4	nowplaying	439
5	anjisalvacion	732
6	puredoctrinesofchrist	831
7	1	1105
8	lolinginlove	1258
9	bbnaija	1405

### 7.2 Differences between *L*- and *K*-embeddings

**Fig 3:**
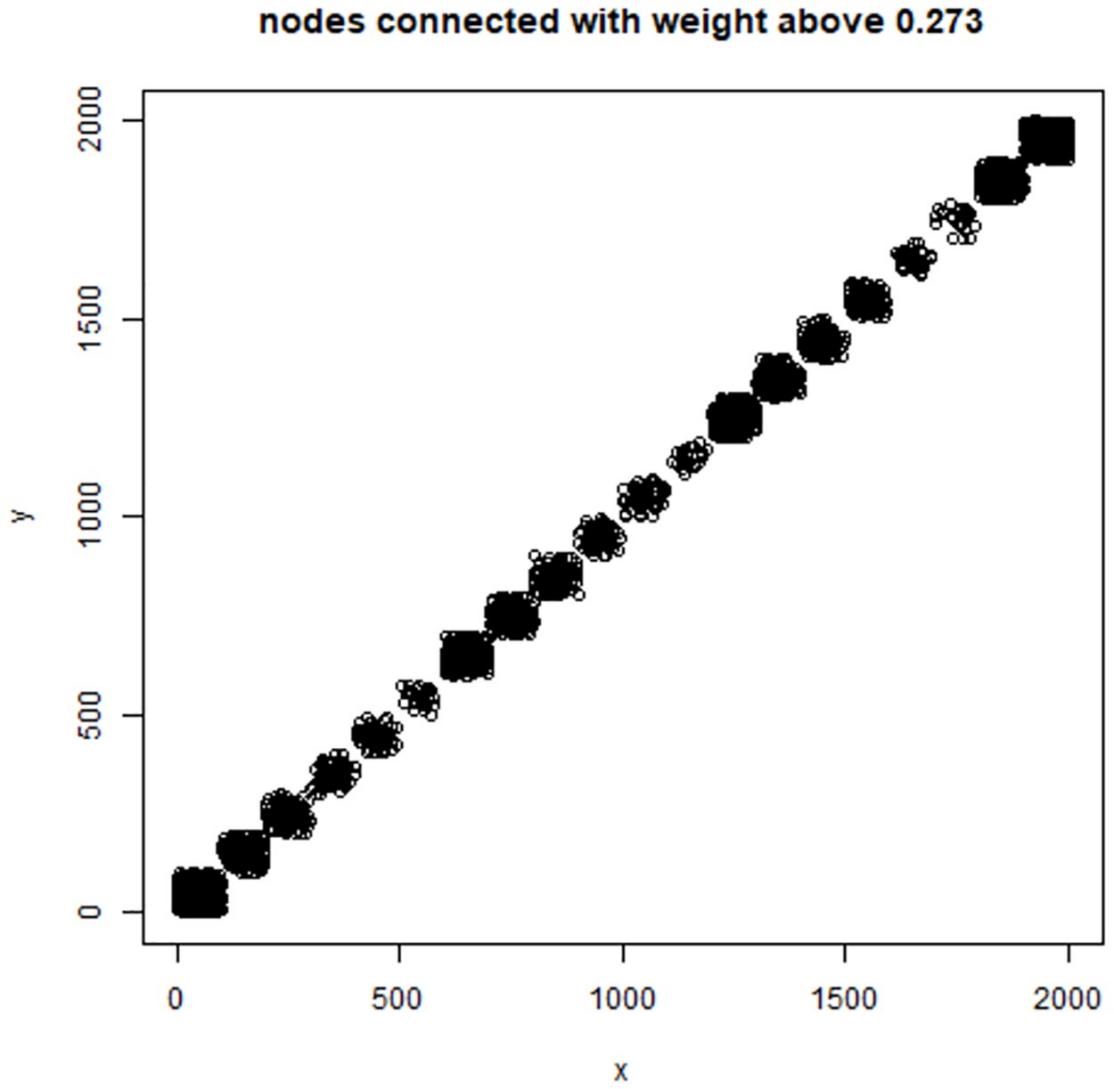
A comparison: Left—Distribution of eigenvalues under *K*-based embedding for BLK.20 data, right—Distribution of eigenvalues under *L*-based embedding for BLK.20 data (20 groups with 100 elements each, the bottom picture).

First, we have computed the spectrograms of the *K*-matrix and *L*-matrix for the TWT.4 dataset. They are shown in Fig 1. One can see that the shapes of these spectrograms differ strongly so that it cannot be claimed that *K*-embedding based clustering and *L*-embedding based clustering rely on related mathematical concepts. Analogous spectrograms have been shown in Figs 2 and 3 for BLK and BLK.20 datasets.

### 7.3 Relationship of *K*-embedding clustering and *L*-embedding clustering

We performed experiments using our synthetic data BLK that creates a block similarity matrix. Such a structure of data is known to be friendly for GSA methodology (it clusters them most successfully).

We clustered the data using the traditional GSA clustering method (*L*-embedding) and using our one (*K*-embedding). The results comparing the clustering produced by each of them are presented in Table 3. In this and in the following confusion matrices, when evaluating the results, we ignore the cluster label permutations and instead consider the correct result being the cell in each column with the maximal cardinality. So the row labels are considered as “ground truth”, while the columns are the “predictors”. In particular, in Table 3, the results of *K*-embedding are taken as “ground truth”.

**Table 3:** Confusion matrix; clsLbased—Clusters generated from *L*-embedding, clsKbased—Clusters generated from *K*-embedding, number of elements in correct clusters: 1913, incorrectly clustered: 87, errors: 4.35%. Parameter: *r* = *number* *of* *clusters* + 1.

clsKbased	clsLbased			
	1	2	3	4
1	0	86	400	0
2	500	0	0	0
3	0	0	0	500
4	0	513	0	1

We see that the clusterings largely agree. We compared also both the “real” groups (that is groups predefined in the data generator) in Tables 4 and 5. We see that *K*-embedding based approach gets closer to real groups than *L*-based approach. This may be due to the fact that *K*-embedding is more similar to *RCut* than *L*-embedding.

**Table 4:** Confusion matrix; clsLbased—Clusters generated from *L*-embedding, clsTrue—The true clusters, number of elements in correct clusters 1899 incorrectly clustered: 101 = errors: 5.05.

clsTrue	clsLbased			
	1	2	3	4
1	500	0	0	0
2	0	0	0	500
3	0	499	0	1
4	0	100	400	0

**Table 5:** Confusion matrix; clsKbased—Clusters generated from *K*-embedding, clsTrue—The true clusters, number of elements in correct clusters: 1984, incorrectly clustered: 16, error: 0.8%.

clsTrue	clsKbased			
	1	2	3	4
1	0	500	0	0
2	0	0	500	0
3	1	0	0	499
4	485	0	0	15

We performed also experiments using real world dataset TWT.4 using both mentioned methods of clustering. The results, comparing the clusterings produced by each of them against the ground truth, being the hashtag groups, are presented in Tables 6 and 7. We see that *K*-embedding based approach gets closer to real groups than *L*-based approach.

**Table 6:** Confusion matrix; clsKbased—Clusters generated from *K*-embedding, clsTrue—The true clusters, number of elements in correct clusters: 1214 incorrectly clustered: 416, errors: 25.52147%.

clsTrue	clsKbased			
	1	2	3	4
#anjisalvacion	370	0	0	10
#lolinginlove	0	0	0	614
#nowplaying	85	27	1	96
#puredoctrinesofchrist	207	0	203	17

**Table 7:** Confusion matrix; clsLbased—Clusters generated from *L*-embedding, clsTrue—The true clusters, number of elements in correct clusters 614 incorrectly clustered: 1016 errors: 62.33129%.

clsTrue	clsLbased			
	1	2	3	4
#anjisalvacion	0	0	0	380
#lolinginlove	1	3	2	608
#nowplaying	0	0	0	209
#puredoctrinesofchrist	0	0	0	427

In summary, we can say that our method gets closer to the intrinsic clustering (that is one indicated by hashtags) than the conventional GSA.

### 7.4 Discrepancies between embeddings and similarities

**Fig 4:**
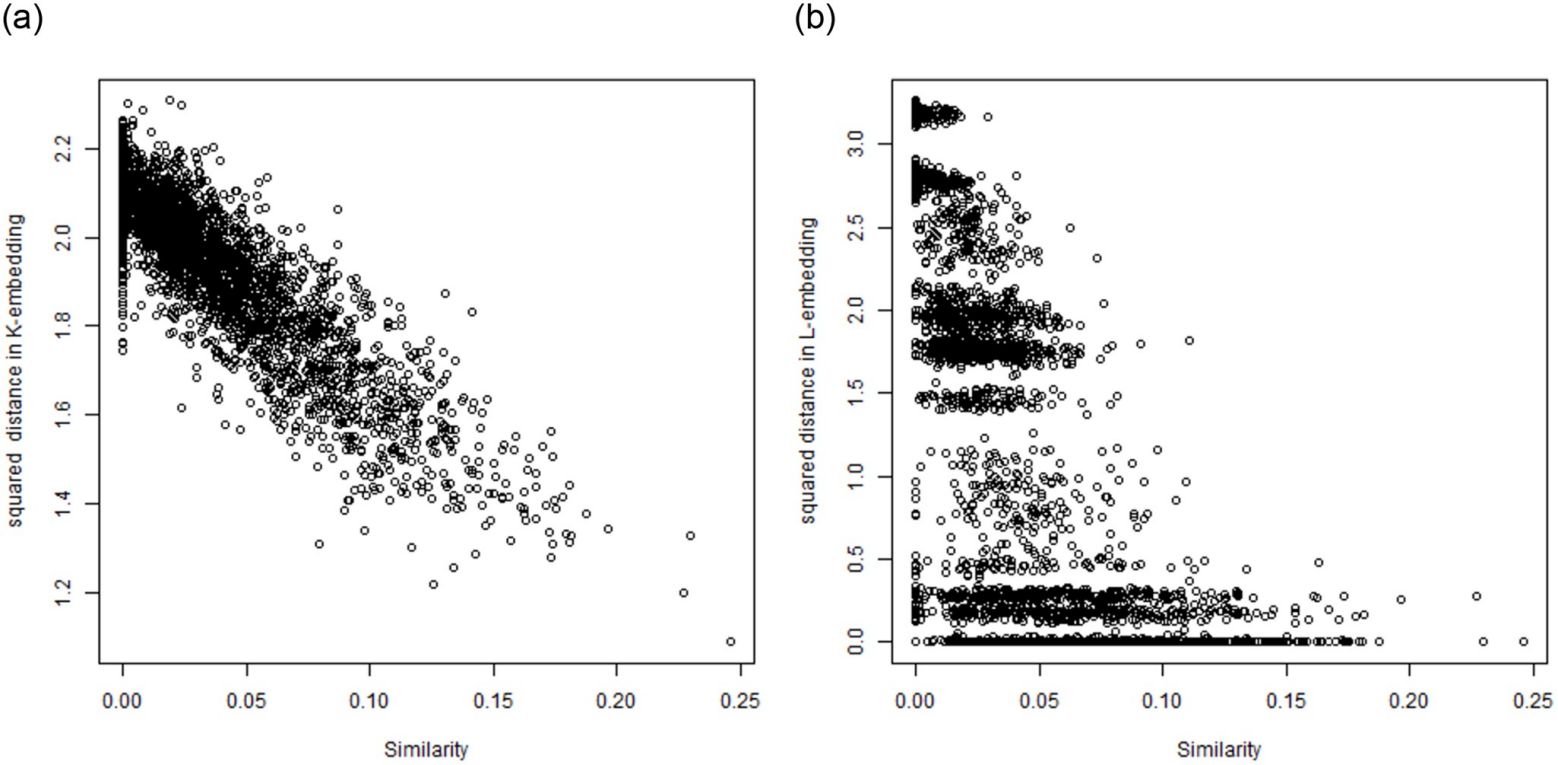
Reconstruction of similarity by squared distance under *K*-embedding with the number of coordinates reduced to 400, BLK dataset (left) and Reconstruction of similarity by squared distance under *L*-embedding with the number of coordinates reduced to 5 (as required by GSA) (right).

**Fig 5:**
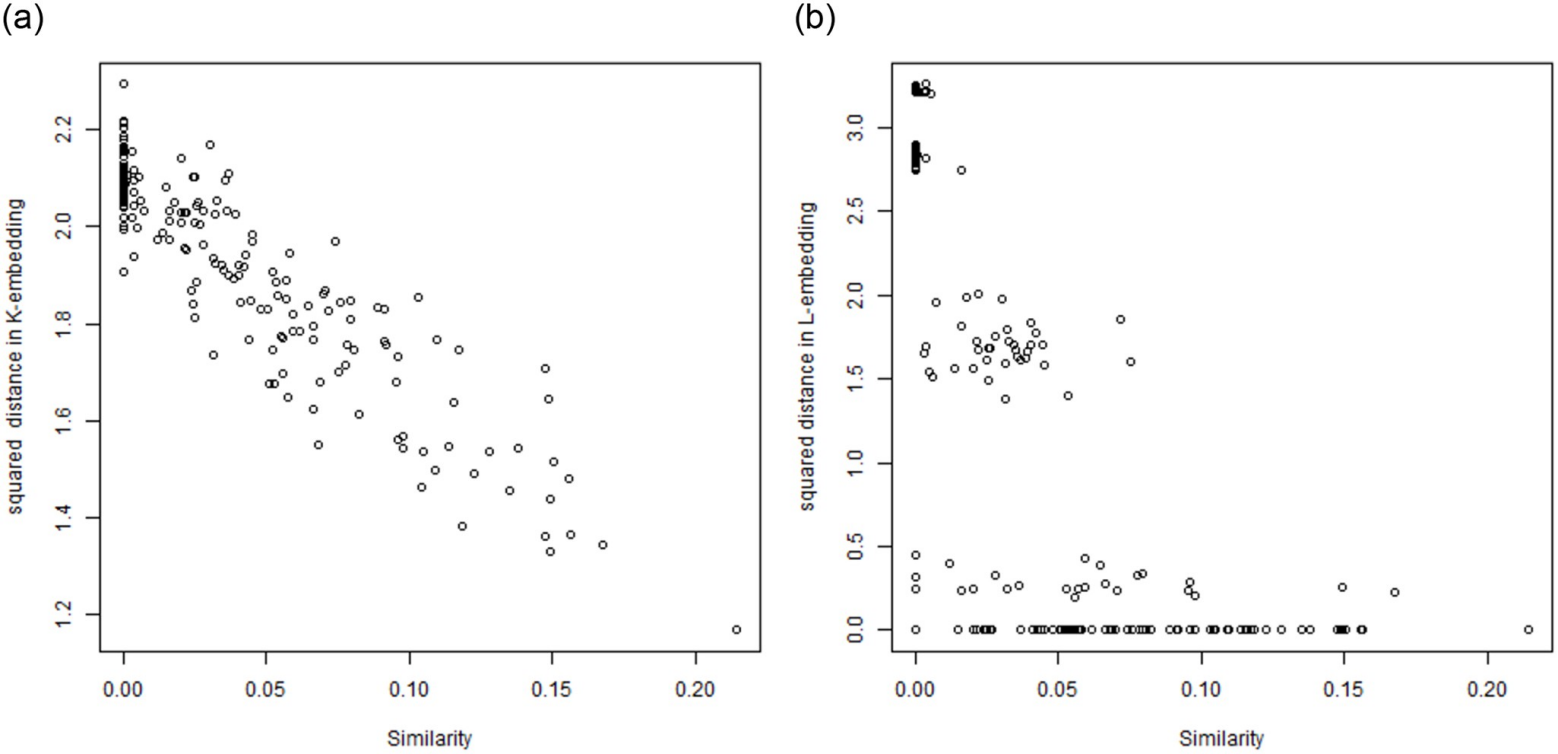
Comparison: Left—Reconstruction of similarity by squared distance under *K*-embedding with the number of coordinates reduced to 400; right—Reconstruction of similarity by squared distance under *L*-embedding with the number of coordinates reduced to 5 (as required by GSA)—For TWT.4 data.

We have also investigated the relationships between the *L*-embedding and the similarities and the *K*-embeddings and similarities for the BLK dataset and the TWT.4 datasets. We randomly selected 80 “documents” and drew in Figs 4 and 5 plots of the distances in the embeddings and the document similarities. One sees that the distances in the *K*-embedding are more closely related to similarities than those of *L*-embeddings. This confirms that we cannot explain the document membership in a cluster based on *L*-embeddings, while *K*-embedding justifies such an interpretation of clusters.

*L*-embedding has the good side that one allows to perform clustering in low dimensions. This may be of course the reason why the relationship of *L*-embedding to similarities is so poor though the *L* matrix itself contains these similarities. In Table 8 we compared the *K* matrix and *L*-matrix reconstruction mean absolute errors when using different numbers of eigenvectors (top eigenvectors for *K*, and low eigenvectors for *L*). As one might have expected, for various numbers of eigenvectors, the reconstruction in *K* is better than in *L*.

**Table 8:** Errors in reconstructing the *K* (errorK) and *L* (errorL) matrix resp. by the subsets of eigenvectors and eigenvalues of various cardinalities (r). They indicate that the *K* reconstruction is better.

r	errorK	errorL
1000	0.001713712	0.03710026
500	0.003453155	0.03710026
250	0.004585440	0.03710026
125	0.005164994	0.03710026
62	0.005363871	0.03710026
31	0.005285830	0.03710026
16	0.005282382	0.03710026
8	0.005252960	0.03710026
4	0.005268428	0.03710026

### 7.5 Clustering performance for 10 hashtags

The experiments were performed on TWT.10 Twitter data for a selected set of 10 hashtags that had to appear only once in the tweet. The hashtags are listed in Table 2. The reason for the choice of such tweets was to have a human-induced reference set on which the quality of clustering was evaluated.

The clustering experiments were performed with popular Python libraries: numpy [47], scipy [48], scikit-learn [49] and soyclustering [50] which is an implementation of spherical *k*-means [51]. In particular, we used

1. SpectralClustering class from scikit-learn with two distinct settings of the affinity parameter: precomputed (affinity from similarity matrix) and nearest_neighbors (affinity from graph of nearest neighbors)—as a representative of the *L*-embedding based clustering (see Table 9)
2. SphericalKMeans class from soyclustering with the following combinations of (init, sparsity) parameter pairs (short names given for reference in Table 11 and following): “sc.n”: (’similar_cut’, None), “sc.sc”: (’similar_cut’, ‘sculley’), “sc.md”: (’similar_cut’, ‘minimum_df’), “k++.n”: (’k-means++’, None), “k++.sc”: (’k-means++’, ‘sculley’), “k++.md”: (’k-means++’, ‘minimum_df’)
3. *K*-embedding clustering (our implementation, exploiting spherical *k*-means). The following numbers of eigenvectors were tried: *r* = 10 (number of hashtags), *r* = 20, *r* = 3577 (half of the tweet count) and *r* = 7155 (tweet count). See Tables 11 and 12.

**Table 9:** *L*-based spectral clustering scores under diverse settings of affinity parameter (column names). All the metrics used are available in the sklearn package, see the documentation at https://scikit-learn.org/stable/api/sklearn.metrics.html.

Score	nearest_neighbors	precomputed
adjusted mutual info score:	**0.486238**	0.400122
adjusted rand score:	**0.308131**	0.139347
completeness score:	**0.394151**	0.268025
fowlkes mallows score	**0.492279**	0.433780
homogeneity score:	0.640522	**0.808333**
mutual info score:	**0.849066**	0.577371
normalized mutual info score:	**0.488004**	0.402568
rand score:	**0.733106**	0.481941
v measure score:	**0.488004**	0.402568
F-score:	**0.1105**	0.0413
F-score average:	**0.0596**	0.0291

As visible from Tables 9 and 10, the spherical *k*-means worst F-score (0.16281 for “k++.n”) is superior to the best spectral clustering score (0.1105 for nearest_neighbors). *K*-embedding based clustering (Tables 11 and 12) best F-score achieved was 0.2144 for “sc.sc” and r = 7155.

**Table 10:** Spherical *k*-means, achieved scores under diverse settings of the algorithm; the highest values in 10 runs.

Score	sc.n	sc.sc	sc.md
adjusted mutual info score	0.442456	**0.481259**	0.450716
adjusted rand score	0.370633	**0.409385**	0.389930
completeness score	0.452317	**0.482101**	0.448153
fowlkes mallows score	0.454743	**0.485733**	0.467060
homogeneity score	0.435729	**0.483157**	0.456226
mutual info score	0.938634	**1.040801**	0.982786
normalized mutual info score	0.443868	**0.482629**	0.452153
rand score	0.854369	**0.867059**	0.865477
v measure score	0.443868	**0.482629**	0.452153
F-score	0.185137	**0.235635**	0.180591
F-score average:	0.0881	**0.1077**	0.1070
Score	k++.n	k++.sc	k++.md
adjusted mutual info score	0.377781	0.430736	0.412699
adjusted rand score	0.352018	0.391106	0.377490
completeness score	0.375782	0.428704	0.414931
fowlkes mallows score	0.433405	0.467857	0.456492
homogeneity score	0.383106	0.435809	0.413615
mutual info score	0.825274	0.938805	0.890996
normalized mutual info score	0.379409	0.432227	0.414272
rand score	0.857969	0.866138	0.862212
v measure score	0.379409	0.432227	0.414272
F-score	0.162810	0.166237	0.198266
F-score average:	0.0937	0.0765	0.0974

**Table 11:** *K*-based clustering, scores (rows) under diverse settings of spherical *k*-means algorithm (columns), when the number of dimensions used (columns) r = 10; the highest values in 10 runs.

Score	sc.n	sc.sc	sc.md
adjusted mutual info score	0.259935	0.273119	0.259381
adjusted rand score	**0.261349**	0.241035	0.236007
completeness score	0.260018	0.268650	0.256508
fowlkes mallows score	**0.362643**	0.352751	0.346052
homogeneity score	0.263817	0.281839	0.266431
mutual info score	0.560122	0.578717	0.552560
normalized mutual info score	0.261904	0.275087	0.261375
rand score	**0.825399**	0.810171	0.812104
v measure score	0.261904	0.275087	0.261375
F-score	0.1635	0.1335	0.1348
F-score average:	0.1005	0.0904	0.0757
Score	k++.n	k++.sc	k++.md
adjusted mutual info score	**0.282358**	0.255432	0.259865
adjusted rand score	0.252918	0.238203	0.241569
completeness score	**0.278569**	0.253377	0.257751
fowlkes mallows score	0.360486	0.347467	0.349507
homogeneity score	**0.290259**	0.261615	0.266085
mutual info score	**0.600085**	0.545816	0.555239
normalized mutual info score	**0.284294**	0.257430	0.261852
rand score	0.816440	0.813288	0.815278
v measure score	**0.284294**	0.257430	0.261852
F-score	**0.2119**	0.1624	0.1396
F-score average:	0.0850	**0.1048**	0.0861

**Table 12:** *K*-based clustering, scores (rows) under diverse settings of spherical *k*-means algorithm (columns), when the number of dimensions used (columns) r = 7155; the highest values in 10 runs.

Score	sc.n	sc.sc	sc.md
adjusted mutual info score	0.351894	0.385909	0.284980
adjusted rand score	0.331422	0.357056	0.285706
completeness score	0.356581	0.391112	0.288709
fowlkes mallows score	0.417609	0.439716	0.378092
homogeneity score	0.350650	0.383984	0.285026
mutual info score	0.768135	0.842521	0.621927
normalized mutual info score	0.353591	0.387516	0.286856
rand score	0.849867	0.855971	0.839133
v measure score	0.353591	0.387516	0.286856
F-score	0.1051	**0.2144**	0.1317
F-score average:	0.0662	0.0930	0.0757
Score	k++.n	k++.sc	k++.md
adjusted mutual info score	**0.401542**	0.360389	0.356130
adjusted rand score	**0.377350**	0.340112	0.335164
completeness score	**0.408082**	0.364872	0.360456
fowlkes mallows score	**0.456293**	0.425090	0.420925
homogeneity score	0.398242	**0.359300**	0.355218
mutual info score	**0.879077**	0.785996	0.776483
normalized mutual info score	**0.403102**	0.362065	0.357818
rand score	**0.862309**	0.851955	0.850621
v measure score	**0.403102**	0.362065	0.357818
F-score	0.1864	0.2015	0.1642
F-score average:	0.0913	0.0933	**0.1000**

This experiment demonstrates that *K*-embedding clustering can approximate a real-world data clustering at a level at least comparable with *L*-based clustering, so that the clustering explanation bridge *L*-embedding—*K*-embedding—Term Vector Space embedding appears to be justified.

Finally, we have checked which clustering results are closer to those of *K*-based clustering—it turned out that spherical clustering is closer than spectral one, see Table 13.

**Table 13:** Best F-score for predicting spectral clustering and spherical clustering by *K*-based clustering for r = 3754.

config	F1 for spectral		F1 for spherical
	precomp	nn	
sc.n	0.0604	0.1129	**0.1820**
sc.sc	0.1101	0.1343	**0.1422**
sc.md	0.1315	0.1469	**0.1627**
k++.n	0.0940	0.1042	**0.1358**
k++.sc	0.0612	0.1359	**0.2608**
k++.md	0.1034	0.1754	**0.1817**

### 7.6 A discussion

The selected real-world dataset TWT.10 was in general not friendly for spectral clustering methods. Nonetheless, it points out that our *K*-embedding can be a candidate for substitution of *L*-embedding for such cases. On the other hand, the GSA friendly (artificial) dataset supports our claim that the explanation path that we have proposed is justified.

**Fig 6:**
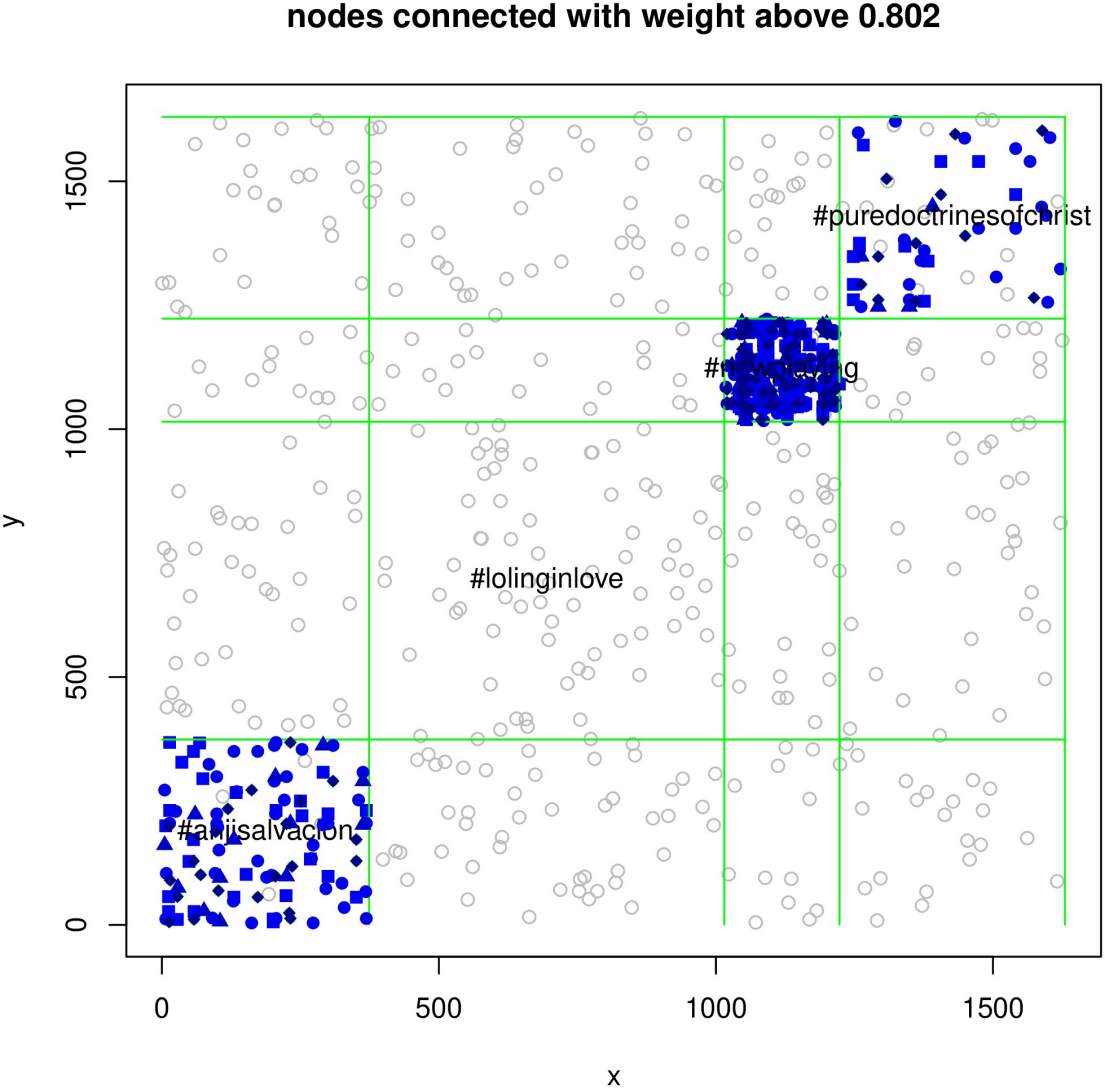
Similarities within and between hashtag related documents. Gray squares: 400 lowest similarities between the documents. Blue squares: top similarities between documents.

**Fig 7:**
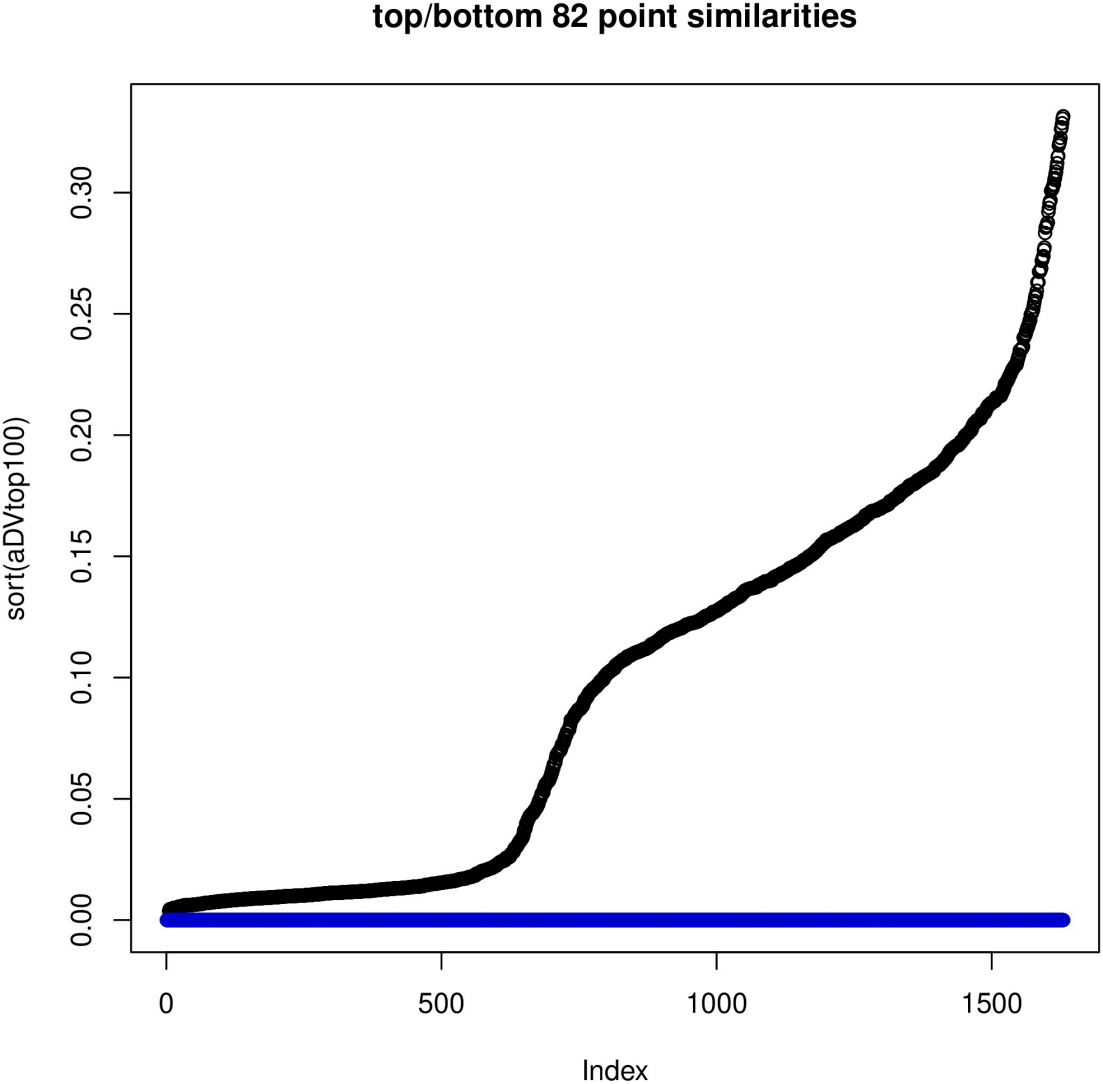
Average top similarities and bottom similarities.

**Fig 8:**
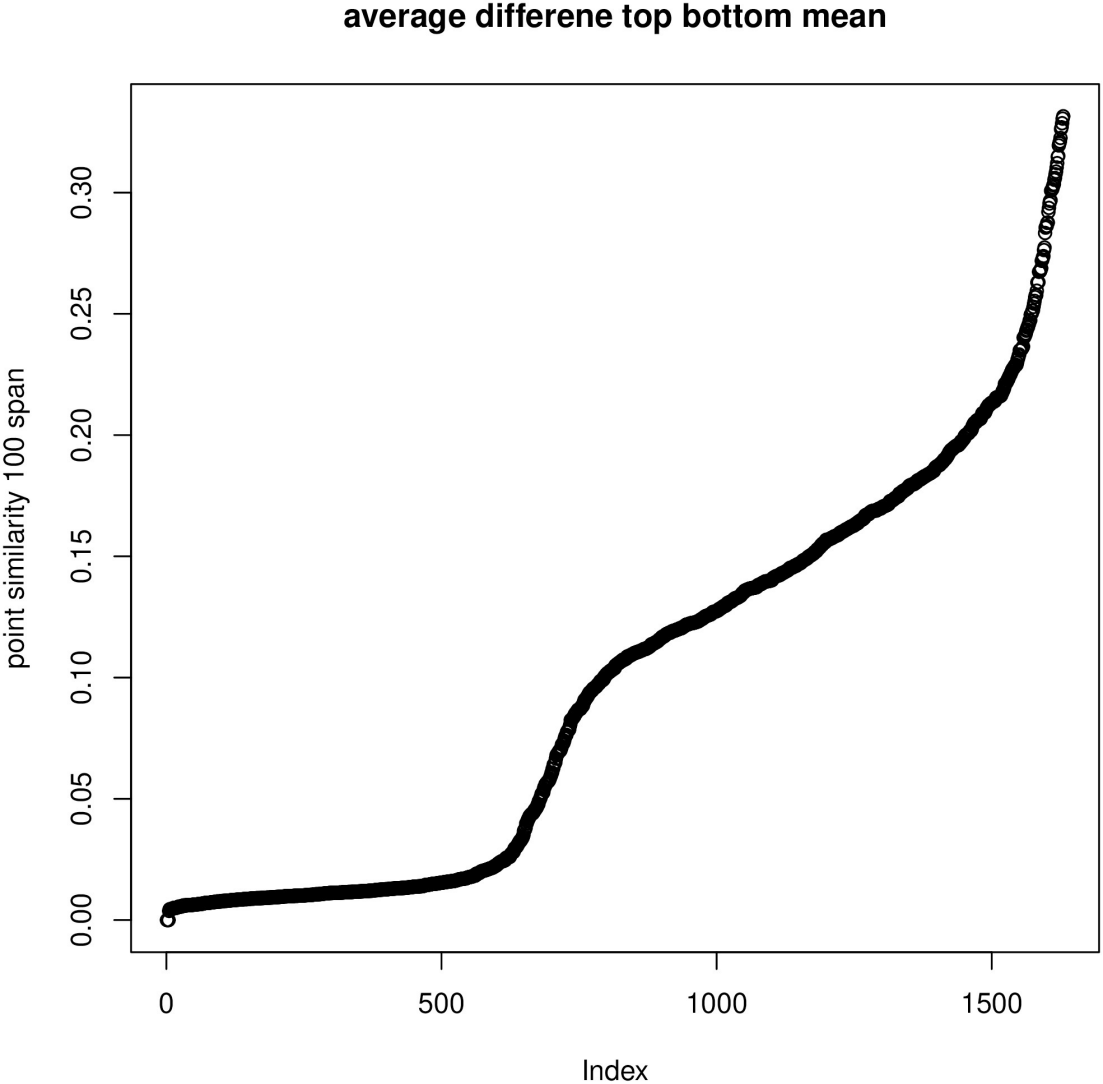
Differences between average top similarities and bottom similarities.

In order to deepen the understanding of the reasons for this observation, please have a look at the following Figs 6–8. It is known from the literature that GSA works best when the similarity matrix between documents has a block-matrix structure. Fig 6 shows that this is not necessarily the case with the data that we are working with. First of all, the top similarities between documents, though located within groups of documents sharing a hashtag, are not evenly distributed over various hashtags. There are even hashtags without any top similarity values. On the other hand, low similarities between documents are not only present outside of hashtag groups, but are also quite common within these groups. Therefore guessing what is the right number of clusters from the similarity distribution is quite hard.

Fig 7 illustrates this point from another perspective. Average of 5% of top similarities were computed for each document (black line); Also average of 5% of bottom similarities was computed for each document (blue line). One sees that there are numerous documents that have not a big span between top and bottom similarities (see also Fig 8). So their cluster membership may be deemed questionable and their assignment to concrete clusters a bit random.

**Fig 9:**
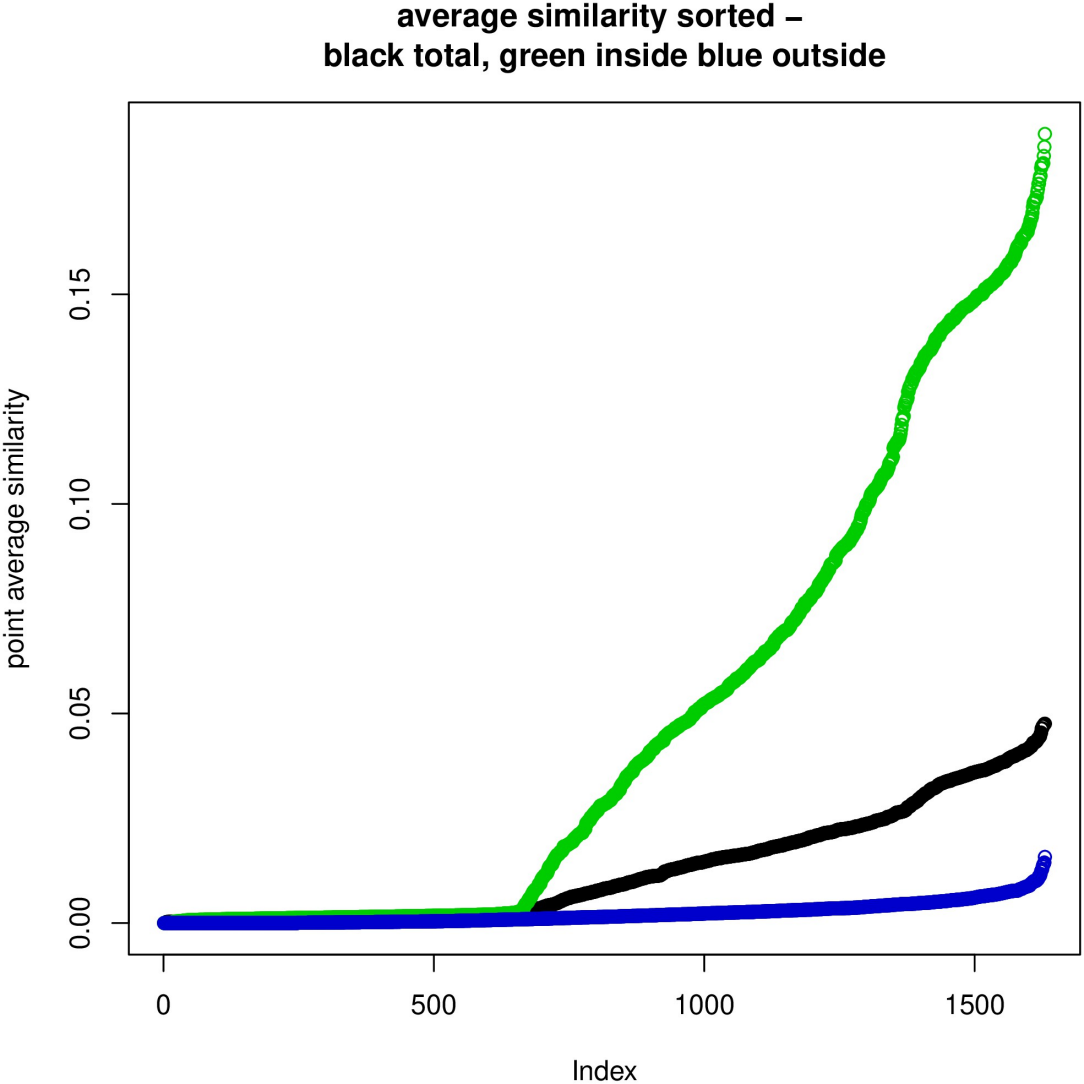
Average similarity of a document: General—Black, within a cluster—Green, between clusters—Blue.

This point is also visible in Fig 9. We see there that for a considerable amount of documents, their similarities within the same hashtag group and outside do not differ significantly.

Last but not least a manual inspection of some of the hashtags suggests that their content is not a real content but the result of some more or less random or mysterious word assignment.

## 8 Conclusions

We have constructed a theoretical bridge linking the clusters resulting from Graph Spectral Clustering and the actual document content, given that similarities between documents are computed as cosine measures in tf (term frequency) or tfidf (term frequency-inverse document frequency) representation. This link enables to provide with explanation of cluster membership in clusters produced by GSA. We provide textual justification for a document’s cluster membership derived from cosine similarity, and at the same time provide textual justification for its non-membership in other clusters. via distance computation in the document vector embedding space.

This result is novel as various authors recommend not to use GSA if you “need an explainable algorithm”. See e.g. https://crunchingthedata.com/when-to-use-spectral-clustering/.

Our result is based on a comparative study of three different embeddings of documents: one in the Term Vector Space, one in the spectral clustering space and one based on the kernel approach. The kernel-based approach shares with the Term Vector Space approach the reproduction of cosine similarity while performing traditional *k*-means clustering, but has much lower dimensionality. On the other hand, both kernel-based approach and spectral analysis based approach use the traditional (distance-based) *k*-means at their heart and approximate the same target function. We have investigated both combinatorial and normalized Laplacian based clustering methods along this path. By comparing the theoretical results we get a new insight into the difference between both types of GSA. While combinatorial Laplacian induces the plain explanation via distances in the Term Vector Space, the explanation for the normalized Laplacian leads us to usage of weighted versions of document embeddings in the Term Vector Space. This can be also considered as a novel insight. So the bridge we have established can be used not only as explanation of cluster membership but also as an insight into the GSA methodology itself.

An important question for future research is the issue under what conditions some number of clusters have been chosen. There has been some research on the automated selection of the number of clusters in general [52] and in spectral clustering domain [53], but the results for real data that were available to us were not satisfactory so this topic should be considered as a future research area.

## 9 Appendix

### 9.1 *L*-based clustering versus *K*-based clustering

Experiments with artificial data were performed, investigating the methodology of Sec. 5.1.

An artificial dataset with 20 clusters (of equal cardinality 100) was generated (in the same way as the BLK.4 dataset). The profile of this dataset in terms of eigenvalues in the L-based and K-based clustering method is shown in Fig 3.

The clustering results of both methods are compared in Tables 14–16.

**Table 14:** Does L-based GSC imply our K-based GSC? Number of elements in correct clusters: 1936, incorrectly clustered: 64 = errors: 3.2%.

TRUE/PRED	1	2	3	4	5	6	7	8	9	10	11	12	13	14	15	16	17	18	19	20
1	0	100	0	0	0	0	0	0	0	0	0	0	0	0	0	0	0	0	0	0
2	0	0	0	0	0	0	0	0	0	0	0	20	0	0	0	0	0	0	0	0
3	0	0	0	56	0	0	0	0	0	0	0	0	0	0	0	0	0	0	0	0
4	100	0	0	0	0	0	0	100	0	0	0	0	0	0	0	0	0	0	0	0
5	0	0	0	0	100	0	0	0	0	0	0	0	0	0	0	0	0	0	0	0
6	0	0	0	0	0	0	0	0	0	0	0	0	0	0	100	100	0	0	0	0
7	0	0	0	0	0	0	0	0	0	0	100	0	0	0	0	0	0	0	0	0
8	0	0	0	0	0	0	0	0	0	100	0	0	0	0	0	0	0	0	0	0
9	0	0	0	0	0	0	0	0	0	0	0	0	0	0	0	0	100	0	0	0
10	0	0	0	0	0	0	100	0	0	0	0	0	0	0	0	0	0	0	0	0
11	0	0	0	0	0	0	0	0	0	0	0	0	100	0	0	0	0	0	0	0
12	0	0	0	44	0	0	0	0	0	0	0	0	0	0	0	0	0	0	0	0
13	0	0	0	0	0	0	0	0	0	0	0	0	0	100	0	0	0	0	0	0
14	0	0	0	0	0	0	0	0	0	0	0	0	0	0	0	0	0	0	100	0
15	0	0	0	0	0	0	0	0	0	0	0	0	0	0	0	0	0	0	0	100
16	0	0	100	0	0	0	0	0	0	0	0	0	0	0	0	0	0	0	0	0
17	0	0	0	0	0	0	0	0	0	0	0	0	0	0	0	0	0	100	0	0
18	0	0	0	0	0	0	0	0	100	0	0	0	0	0	0	0	0	0	0	0
19	0	0	0	0	0	100	0	0	0	0	0	0	0	0	0	0	0	0	0	0
20	0	0	0	0	0	0	0	0	0	0	0	80	0	0	0	0	0	0	0	0

**Table 15:** Does our K-based method imply L-based GSC? Number of elements in correct clusters: 1800, incorrectly clustered: 200 = errors: 10%.

TRUE/PRED	1	2	3	4	5	6	7	8	9	10	11	12	13	14	15	16	17	18	19	20
1	0	0	0	100	0	0	0	0	0	0	0	0	0	0	0	0	0	0	0	0
2	100	0	0	0	0	0	0	0	0	0	0	0	0	0	0	0	0	0	0	0
3	0	0	0	0	0	0	0	0	0	0	0	0	0	0	0	100	0	0	0	0
4	0	0	56	0	0	0	0	0	0	0	0	44	0	0	0	0	0	0	0	0
5	0	0	0	0	100	0	0	0	0	0	0	0	0	0	0	0	0	0	0	0
6	0	0	0	0	0	0	0	0	0	0	0	0	0	0	0	0	0	0	100	0
7	0	0	0	0	0	0	0	0	0	100	0	0	0	0	0	0	0	0	0	0
8	0	0	0	100	0	0	0	0	0	0	0	0	0	0	0	0	0	0	0	0
9	0	0	0	0	0	0	0	0	0	0	0	0	0	0	0	0	0	100	0	0
10	0	0	0	0	0	0	0	100	0	0	0	0	0	0	0	0	0	0	0	0
11	0	0	0	0	0	0	100	0	0	0	0	0	0	0	0	0	0	0	0	0
12	0	20	0	0	0	0	0	0	0	0	0	0	0	0	0	0	0	0	0	80
13	0	0	0	0	0	0	0	0	0	0	100	0	0	0	0	0	0	0	0	0
14	0	0	0	0	0	0	0	0	0	0	0	0	100	0	0	0	0	0	0	0
15	0	0	0	0	0	100	0	0	0	0	0	0	0	0	0	0	0	0	0	0
16	0	0	0	0	0	100	0	0	0	0	0	0	0	0	0	0	0	0	0	0
17	0	0	0	0	0	0	0	0	100	0	0	0	0	0	0	0	0	0	0	0
18	0	0	0	0	0	0	0	0	0	0	0	0	0	0	0	0	100	0	0	0
19	0	0	0	0	0	0	0	0	0	0	0	0	0	100	0	0	0	0	0	0
20	0	0	0	0	0	0	0	0	0	0	0	0	0	0	100	0	0	0	0	0

**Table 16:** Is true clustering implied with our K-based method? Number of elements in correct clusters: 1800, incorrectly clustered: 200 = errors: 10%.

TRUE/PRED	1	2	3	4	5	6	7	8	9	10	11	12	13	14	15	16	17	18	19	20
1	0	0	0	0	0	0	0	0	0	0	0	0	0	0	0	0	100	0	0	0
2	0	0	0	100	0	0	0	0	0	0	0	0	0	0	0	0	0	0	0	0
3	0	0	0	0	100	0	0	0	0	0	0	0	0	0	0	0	0	0	0	0
4	0	0	0	0	0	0	0	100	0	0	0	0	0	0	0	0	0	0	0	0
5	0	0	0	0	0	100	0	0	0	0	0	0	0	0	0	0	0	0	0	0
6	0	0	0	0	0	100	0	0	0	0	0	0	0	0	0	0	0	0	0	0
7	0	0	0	0	0	0	0	0	0	0	100	0	0	0	0	0	0	0	0	0
8	0	0	0	100	0	0	0	0	0	0	0	0	0	0	0	0	0	0	0	0
9	0	0	0	0	0	0	0	0	0	0	0	0	0	0	0	100	0	0	0	0
10	0	0	0	0	0	0	0	0	100	0	0	0	0	0	0	0	0	0	0	0
11	0	20	0	0	0	0	0	0	0	0	0	0	0	0	0	0	0	0	0	80
12	100	0	0	0	0	0	0	0	0	0	0	0	0	0	0	0	0	0	0	0
13	0	0	0	0	0	0	0	0	0	100	0	0	0	0	0	0	0	0	0	0
14	0	0	0	0	0	0	100	0	0	0	0	0	0	0	0	0	0	0	0	0
15	0	0	0	0	0	0	0	0	0	0	0	0	0	100	0	0	0	0	0	0
16	0	0	0	0	0	0	0	0	0	0	0	0	0	0	0	0	0	0	100	0
17	0	0	0	0	0	0	0	0	0	0	0	0	100	0	0	0	0	0	0	0
18	0	0	0	0	0	0	0	0	0	0	0	0	0	0	100	0	0	0	0	0
19	0	0	0	0	0	0	0	0	0	0	0	0	0	0	0	0	0	100	0	0
20	0	0	56	0	0	0	0	0	0	0	0	44	0	0	0	0	0	0	0	0

### 9.2 *N*-based clustering versus M-based clustering

Again, experiments with artificial data were performed, investigating the methodology of Sec. 6.1.

An artificial dataset with 20 clusters (of equal cardinality 100) was generated (in the same way as the BLK.4 dataset).

The clustering results of both methods are compared in Tables 17–19.

**Table 17:** Does L-based GSC imply our M-based GSC? Number of elements in correct clusters: 1900, incorrectly clustered: 100 = errors: 5%.

TRUE/PRED	1	2	3	4	5	6	7	8	9	10	11	12	13	14	15	16	17	18	19	20
1	0	0	0	0	0	0	0	0	0	0	0	0	0	0	0	100	0	0	0	0
2	0	100	0	0	0	0	0	0	0	0	0	0	0	0	0	0	0	0	0	0
3	0	0	0	0	0	0	0	0	100	0	0	0	0	0	0	0	0	0	0	0
4	0	0	0	0	0	0	0	0	0	0	0	0	0	0	100	0	0	0	0	0
5	0	0	0	0	0	0	0	0	0	0	0	99	0	0	0	0	0	0	0	0
6	0	0	0	0	0	0	0	0	0	0	0	0	0	0	0	0	100	0	0	0
7	0	0	0	0	0	0	0	0	0	0	0	1	0	0	0	0	0	0	0	100
8	0	0	0	0	0	0	0	0	0	0	0	100	0	0	0	0	0	0	0	0
9	0	0	0	0	0	0	0	100	0	0	0	0	0	0	0	0	0	0	0	0
10	0	0	0	0	0	0	0	0	0	0	0	0	100	0	0	0	0	0	0	0
11	0	0	0	0	0	0	0	0	0	0	0	0	0	0	0	0	0	100	0	0
12	0	0	0	0	0	0	0	0	0	100	0	0	0	0	0	0	0	0	0	0
13	0	0	0	0	0	0	0	0	0	0	0	0	0	0	0	0	0	0	100	0
14	100	0	0	0	0	0	0	0	0	0	0	0	0	0	0	0	0	0	0	0
15	0	0	0	0	100	0	0	0	0	0	0	0	0	0	0	0	0	0	0	0
16	0	0	58	0	0	42	0	0	0	0	0	0	0	0	0	0	0	0	0	0
17	0	0	0	0	0	0	0	0	0	0	100	0	0	0	0	0	0	0	0	0
18	0	0	0	0	0	0	0	0	0	0	0	0	0	100	0	0	0	0	0	0
19	0	0	0	0	0	0	100	0	0	0	0	0	0	0	0	0	0	0	0	0
20	0	0	0	100	0	0	0	0	0	0	0	0	0	0	0	0	0	0	0	0

**Table 18:** Is true clustering implied with our M-based method? Number of elements in correct clusters: 1999, incorrectly clustered: 1 = errors: 0.05%.

TRUE/PRED	1	2	3	4	5	6	7	8	9	10	11	12	13	14	15	16	17	18	19	20
1	0	0	0	0	0	0	0	0	0	0	0	0	100	0	0	0	0	0	0	0
2	0	0	100	0	0	0	0	0	0	0	0	0	0	0	0	0	0	0	0	0
3	0	0	0	0	0	0	100	0	0	0	0	0	0	0	0	0	0	0	0	0
4	0	0	0	0	0	0	0	0	0	0	0	100	0	0	0	0	0	0	0	0
5	0	0	0	0	0	0	0	0	0	0	0	0	0	0	0	0	100	0	0	0
6	0	0	0	0	0	0	0	0	0	0	100	0	0	0	0	0	0	0	0	0
7	0	0	0	0	0	0	0	0	0	0	0	0	0	0	0	0	0	100	0	0
8	0	0	0	0	0	0	0	0	0	0	0	0	0	0	0	100	0	0	0	0
9	0	0	0	0	0	100	0	0	0	0	0	0	0	0	0	0	0	0	0	0
10	0	0	0	0	99	0	1	0	0	0	0	0	0	0	0	0	0	0	0	0
11	0	0	0	0	0	0	0	100	0	0	0	0	0	0	0	0	0	0	0	0
12	0	0	0	0	0	0	0	0	100	0	0	0	0	0	0	0	0	0	0	0
13	0	0	0	0	0	0	0	0	0	0	0	0	0	100	0	0	0	0	0	0
14	0	0	0	0	0	0	0	0	0	0	0	0	0	0	0	0	0	0	0	100
15	0	0	0	100	0	0	0	0	0	0	0	0	0	0	0	0	0	0	0	0
16	0	0	0	0	0	0	0	0	0	100	0	0	0	0	0	0	0	0	0	0
17	100	0	0	0	0	0	0	0	0	0	0	0	0	0	0	0	0	0	0	0
18	0	0	0	0	0	0	0	0	0	0	0	0	0	0	0	0	0	0	100	0
19	0	0	0	0	0	0	0	0	0	0	0	0	0	0	100	0	0	0	0	0
20	0	100	0	0	0	0	0	0	0	0	0	0	0	0	0	0	0	0	0	0

**Table 19:** Is true clustering implied with L-based GSC? Number of elements in correct clusters: 1900, incorrectly clustered: 100 = errors: 5%.

TRUE/PRED	1	2	3	4	5	6	7	8	9	10	11	12	13	14	15	16	17	18	19	20
1	0	0	0	0	0	0	0	0	0	0	0	0	0	0	0	0	0	0	100	0
2	0	0	0	0	0	0	0	0	100	0	0	0	0	0	0	0	0	0	0	0
3	0	0	0	0	0	0	0	0	0	0	0	0	0	0	0	0	0	0	0	100
4	0	0	0	0	0	0	0	0	0	100	0	0	0	0	0	0	0	0	0	0
5	0	0	0	0	0	0	0	0	0	0	100	0	0	0	0	0	0	0	0	0
6	0	0	0	0	0	0	0	0	0	0	0	0	0	0	0	0	0	100	0	0
7	0	0	0	0	0	0	0	0	0	0	0	0	0	100	0	0	0	0	0	0
8	0	0	58	0	0	42	0	0	0	0	0	0	0	0	0	0	0	0	0	0
9	0	0	0	0	0	0	0	0	0	0	0	0	0	0	0	0	100	0	0	0
10	0	0	0	0	0	0	0	0	0	0	0	100	0	0	0	0	0	0	0	0
11	0	0	0	0	0	0	0	0	0	0	0	100	0	0	0	0	0	0	0	0
12	0	0	0	0	0	0	0	100	0	0	0	0	0	0	0	0	0	0	0	0
13	100	0	0	0	0	0	0	0	0	0	0	0	0	0	0	0	0	0	0	0
14	0	0	0	100	0	0	0	0	0	0	0	0	0	0	0	0	0	0	0	0
15	0	0	0	0	0	0	0	0	0	0	0	0	0	0	100	0	0	0	0	0
16	0	0	0	0	0	0	0	0	0	0	0	0	100	0	0	0	0	0	0	0
17	0	0	0	0	0	0	0	0	0	0	0	0	0	0	0	100	0	0	0	0
18	0	0	0	0	0	0	100	0	0	0	0	0	0	0	0	0	0	0	0	0
19	0	0	0	0	100	0	0	0	0	0	0	0	0	0	0	0	0	0	0	0
20	0	100	0	0	0	0	0	0	0	0	0	0	0	0	0	0	0	0	0	0

We asked also: Is true clustering implied with L-based GSC? perfect match of clusterings was observed.
